# GPUs Outperform Current HPC and Neuromorphic Solutions in Terms of Speed and Energy When Simulating a Highly-Connected Cortical Model

**DOI:** 10.3389/fnins.2018.00941

**Published:** 2018-12-12

**Authors:** James C. Knight, Thomas Nowotny

**Affiliations:** Centre for Computational Neuroscience and Robotics, School of Engineering and Informatics, University of Sussex, Brighton, United Kingdom

**Keywords:** GPU, high-performance computing, parallel computing, accuracy of simulation, energy to solution, benchmarking, computational neuroscience, spiking neural networks

## Abstract

While neuromorphic systems may be the ultimate platform for deploying spiking neural networks (SNNs), their distributed nature and optimization for specific types of models makes them unwieldy tools for developing them. Instead, SNN models tend to be developed and simulated on computers or clusters of computers with standard von Neumann CPU architectures. Over the last decade, as well as becoming a common fixture in many workstations, NVIDIA GPU accelerators have entered the High Performance Computing field and are now used in 50 % of the Top 10 super computing sites worldwide. In this paper we use our GeNN code generator to re-implement two neo-cortex-inspired, circuit-scale, point neuron network models on GPU hardware. We verify the correctness of our GPU simulations against prior results obtained with NEST running on traditional HPC hardware and compare the performance with respect to speed and energy consumption against published data from CPU-based HPC and neuromorphic hardware. A full-scale model of a cortical column can be simulated at speeds approaching 0.5× real-time using a single NVIDIA Tesla V100 accelerator—faster than is currently possible using a CPU based cluster or the SpiNNaker neuromorphic system. In addition, we find that, across a range of GPU systems, the energy to solution as well as the energy per synaptic event of the microcircuit simulation is as much as 14× lower than either on SpiNNaker or in CPU-based simulations. Besides performance in terms of speed and energy consumption of the simulation, efficient initialization of models is also a crucial concern, particularly in a research context where repeated runs and parameter-space exploration are required. Therefore, we also introduce in this paper some of the novel parallel initialization methods implemented in the latest version of GeNN and demonstrate how they can enable further speed and energy advantages.

## 1. Introduction

Currently, the most common way to accelerate large-scale spiking neural network (SNN) simulations is to use CPU-based HPC clusters running software simulators such as NEST (Gewaltig and Diesmann, 2007) or parallel Neuron (Carnevale and Hines, 2006). However, CPU-based systems are not well-suited to exploiting the large amounts of fine-grained parallelism present in SNN simulations. Furthermore, in order to reduce simulation times, models must be spread across large numbers of compute nodes meaning that performance is ultimately constrained by the latency of the MPI interconnect.

Neuromorphic systems use dedicated hardware, inspired by aspects of the brain, to address the problems of parallelism and efficient spike communication. The SpiNNaker system (Furber et al., 2014), developed as part of the Human Brain project (HBP) in Manchester, is a neuromorphic computer consisting of up to a million ARM cores, connected with an interconnect topology optimized for spike-like communication. The BrainScaleS system developed within HBP at Heidelberg (Schemmel et al., 2017), uses analog circuit elements rather than digital processors to emulate the dynamics of point neurons. Spikes then propagate between these circuit elements through a digital interconnect network. Other neuromorphic systems based on various combinations of digital and analog hardware include the Loihi chip (Davies et al., 2018) developed by Intel, the TrueNorth chip (Merolla et al., 2014) built by IBM and the Dynapse system (Qiao et al., 2015) developed at University of Zurich.

While neuromorphic systems offer significant theoretical advantages in terms of power efficiency and simulation speed, this often comes at the expense of flexibility. In systems where physical circuit elements are used to model individual neurons and synapses, the most obvious restriction is that the physical circuits dictate what neuron and synapse models are supported. Furthermore, in neuromorphic systems of this type, these circuits are instantiated in a fixed ratio (for example 64 k synapses to 256 neurons) meaning that Liebig's law dictates that their scalability is limited by the availability of the scarcest of these circuits. Even fully-programmable systems such as SpiNNaker suffer from this issue as, for example, handling high incoming spike rates consumes a large number of CPU cycles, reducing the number of neurons that can be simulated on each core. Some of these issues are illustrated in a recent publication by van Albada et al. (2018) who investigated the comparative performance of simulations of a micro-column model (Potjans and Diesmann, 2014) in NEST-based simulations on an HPC cluster and an implementation on the SpiNNaker neuromorphic system. This model required smaller simulation timesteps and denser connectivity than SpiNNaker was designed for, meaning that, although SpiNNaker achieved the same accuracy as the HPC system, it had to be run 20× slower than realtime with only a small number of neurons simulated on each core. Running the model this slowly meant that the theoretical energy and performance advantages of using the SpiNNaker system—which had been previously demonstrated using models more specifically tuned to its characteristics (Sharp et al., 2012, 2014; Knight et al., 2016)—were lost and the model not only ran faster on the HPC system but also consumed less energy.

Besides measuring the performance in terms of simulation speed, (van Albada et al., 2018) also identified that efficiently **initializing** and loading large-scale models onto neuromorphic systems remains a computational challenge. For example, the cortical microcircuit model developed by Potjans and Diesmann (2014) took 10 h to initialize and load onto SpiNNaker. This confirms earlier observations (Diamond et al., 2016) that prototype neuromorphic systems are not efficient at accelerating their initialization: Both SpiNNaker and a previous generation of the BrainScaleS system spent a significant amount of time and energy initializing network models on a host machine.

These factors suggest that when **developing** SNNs, more flexible accelerators which can accelerate the construction, initialization and simulation of large-scale SNNs are required. Field-Programmable Gate Arrays (FPGAs) are devices consisting of a large number of lookup-table based logic blocks, connected using a programmable fabric. FPGAs have been used to build various “hard-wired” SNN accelerators (Moore et al., 2012; Wang and van Schaik, 2018), but Naylor et al. (2013) showed that they can also be used to develop more flexible, programmable accelerators with comparable performance. However, although systems of this sort could theoretically be used to accelerate the construction and initialization of SNNs as well as their simulation, FPGAs are not yet commonplace in workstations and their lack of hardware support for floating point arithmetic makes them ill-suited for simulating some common classes of neuron and synapse models.

Alternatively, GPU architectures are designed for high throughput applications with large amounts of fine-grained parallelism. They replace the large coherent caches, relied upon by modern CPU architectures to improve performance, with large numbers of floating point arithmetic units connected to high-bandwidth external memory. Programmable GPUs were originally developed to accelerate the rendering of 3D graphics which typically involves applying the same, independent computations to each pixel—for example to calculate its illumination. However, GPU acceleration has proven to be also very useful for accelerating many other tasks, including the training of deep learning systems, and GPUs are now used extensively in modern AI systems. The application of GPU acceleration to SNN simulations is also promising and there are a number of active SNN simulator projects which target GPUs. CARLsim (Chou et al., 2018) is a C++ based simulator using NVIDIA CUDA (Compute Unified Device Architecture) but, as CARLsim is not based on code generation, it is difficult for users without CUDA expertise to add new neuron and synapse models. EDLUT (Garrido et al., 2011) was initially an event-driven CPU based simulator for SNNs, but has evolved into a hybrid CPU/GPU system with support for both, time- and event-driven models. ANNarchy (Vitay et al., 2015) is a code generation based simulator which translates Python model descriptions into multi-core CPU or GPU code with a focus on hybrid rate- and spiking models. Other simulators that have seen less development in the last 2–4 years include NCS6 (Hoang et al., 2013), Myriad (Rittner and Cleland, 2016), and NeMo (Fidjeland et al., 2009) (see Brette and Goodman (2012) for a review). GeNN (Yavuz et al., 2016) is a code-generation library aimed at facilitating accelerated SNN simulations on GPU hardware. It has been designed to strike a balance between flexibility—allowing users to define their own model neurons and synapses—and efficiency in generating optimized CUDA code for the less obviously parallelisable phases of parallel SNN simulations such as spike propagation.

In this paper we introduce novel methods for parallel initialization of SNNs in the GeNN simulator and investigate the performance of a GPU based simulation of the micro-column network model (Potjans and Diesmann, 2014) using GeNN as well as a model using STDP in a highly connected network (Morrison et al., 2007). We then compare to other recent benchmarks (van Albada et al., 2018) and critically discuss the current state of the art for SNN simulations.

## 2. Material and Methods

### 2.1. GPU Architectures

In this section we will briefly discuss GPU hardware architectures and the Single Instruction Multiple Thread (SIMT) paradigm typically used to program them. All GPU manufacturers (confusingly) use their own terminology but because in this paper we use NVIDIA hardware, we will refer to concepts using NVIDIA's terminology. GPUs built by other manufacturers are relatively similar so that our description below applies to other GPUs after appropriate translation. For example, a “Stream Processor” on an AMD GPU is equivalent to a “CUDA core” on an NVIDIA GPU. Similarily, we will discuss SIMT programming in the context of CUDA because GeNN is implemented using CUDA, but OpenCL is conceptually quite similar.

**Figure 1:**
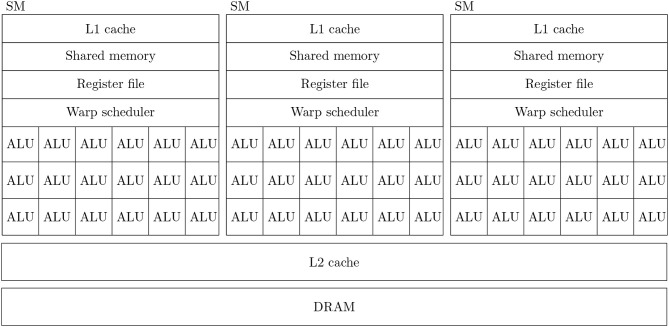
Simplified illustration of an example GPU hardware architecture with 3 streaming multiprocessors.

Figure 1 shows a simplified diagram of the hardware architecture of a typical GPU. As discussed in the introduction, GPUs are designed primarily for high throughput computation and therefore the majority of their die area is used for arithmetic logic units (ALUs) known as *CUDA cores*. Depending on the particular GPU, different CUDA cores might be dedicated to integer, single or double-precision floating point operations. While each CUDA core is independent, they have no capacity for directly executing instructions. Instead they are contained within *Streaming multiprocessors* (SMs) which schedule sequences of Single Instruction Multiple Data (SIMD) instructions known as *warps* to run on the CUDA cores using a piece of hardware known as a *warp scheduler*. The context associated with each active warp is stored in a large register file (64 kB on the Volta architecture) allowing the warp scheduler to very rapidly switch between active warps while they wait for data to be delivered from external memory or for CUDA cores to become available. Therefore, providing that there is sufficient computation to perform, GPUs can effectively hide memory latency by rapidly switching between warps.

In all recent GPU architectures, SMs access the GPU's DRAM through an L2 cache. While modern CPUs typically have 64 bit memory interfaces, modern GPUs have much wider memory interfaces (4096 bit on the Volta architecture). In order to use these wide memory interfaces efficiently, GPU memory controllers aim to combine DRAM accesses made by SMs to adjacent memory addresses into single transactions—a processed known as *coalescing*. Within each SM there is also a small amount (128 kB on the Volta architectures) of much faster local memory which can typically be partitioned by the programmer into software-controlled cache known as *shared memory* and read-only hardware controlled L1 cache.

Efficiently programming SIMD architectures such as SSE or AVX often involves manually inserting intrinisics into serial code to process data in parallel. However, not only is this difficult but, if the underlying architecture and thus the intrinsics which drive it change, applications need to be re-written. NVIDIA CUDA solves this problem by instead presenting the programmer with a more abstract SIMT programming model where programmers write serial code to be executed in parallel across many virtual *threads*. Threads are grouped into *thread blocks* which are scheduled so that they can share data via the shared memory and the thread blocks are grouped into *grids* which represent all the threads required to solve the entire problem. The CUDA compiler and GPU hardware take care of converting this representation into warps of SIMD instructions, scheduling these appropriately and enabling and disabling SIMD lanes within each warp when conditional control flow requires it. For example, adding two vectors x and y of length n could be implemented as follows using CUDA:


__global__ **void** addVec(**int** n, **const float** *x, **float** *y)
{
   **const int** i = (blockIdx. x * blockDim. x) + threadIdx. x;
   **if** (i < n) {
      y[i] += x[i];
   }
}


Aside from the __global__ function decorator which instructs the compiler to hand this function off to CUDA and the blockIdx, blockDim, and threadIdx variables which allow the position of the current thread within the block and grid to be queried, the code is very similar to standard serial C code.

### 2.2. GeNN

As described by Yavuz et al. (2016), GeNN is a code-generation based system that generates model- and platform-optimized CUDA code for GPU accelerated SNN simulations. In doing so, it abstracts the hardware and model dependent code choices mentioned above away from its users. GeNN neuron models are defined by writing a C++ class which defines the model parameters and snippets of C-like code that describe how it should be simulated. For example the following LIF class describes a leaky integrate-and-fire neuron with normalized units, solved algebraically:


**class** LIF:**public** NeuronModels::Base
{
**public**:
   DECLARE_MODEL(LIF,1,1);
   SET_SIM_CODE(“$(V)=($(Isyn)*$(TauM)*(1.0-$(ExpTC)))+($(ExpTC)*
   $(V));\n”);
   SET_THRESHOLD_CONDITION_CODE(“$(V)>=1.0”);
   SET_RESET_CODE(“$(V)=0.0;”);
   SET_PARAM_NAMES({“"TauM”});
   SET_DERIVED_PARAMS({
         {“ExpTC”,[](const vector<**double**> &pars, **double** dt)
                      {**return** exp(−dt/pars[0]);}}});
   SET_VARS({{“V”,“scalar”}});
};
IMPLEMENT_MODEL(LIF);


The DECLARE_MODEL and IMPLEMENT_MODEL macros insert boilerplate code used subsequently for defining parameters and initial model states in a type-safe manner. The SET_SIM_CODE, SET_THRESHOLD_CONDITION_CODE, and SET_RESET_CODE macros specify the snippets of code used, respectively, to update the simulation state, check whether a spike should be emitted and to reset the neuron after a spike. The names of model parameters (constant across the entire population) are specified using the SET_PARAM_NAMES macro and any “pre-processing” logic to be applied to these is specified with SET_DERIVED_PARAMS—in this case converting an exponential decay time constant to a multiplier to be applied every simulation timestep. Finally, the SET_VARS macro specifies the names and types of the per-neuron state variables. These macros provide some “syntactic sugar” but are entirely optional – users can instead override the underlying virtual functions themselves. In GeNN, synapse models are defined using very similar classes with the option to define code snippets for time-driven and event-driven updates. Event-driven updates can be triggered by pre or postsynaptic spikes as well as by custom events, for example the pre or postsynaptic neuron's membrane voltages crossing a threshold. Once the required models have been defined, the values of parameters and initial state variables can be set and *populations* of neurons can be added to a network:


InitVarSnippet::Uniform::ParamValues vDist(0.0,1.0);
LIF::ParamValues params(20.0);
LIF::VarValues initState(initVar<InitVarSnippet::Uniform>(vDist));
network.addNeuronPopulation<LIF>(“pop”,1000, params, initState);


This listing also illustrates how, in the latest version of GeNN, the approach used for defining models can also be used to configure how variables are initialized. In the listing the membrane voltage V of our 1,000 LIF neurons is sampled from the uniform distribution using one of GeNN's built in *variable initialization snippets*. These are definied in a similar manner to the neuron model presented earlier in this section and, by using this mechanism, GeNN can offload network initialization to the GPU using the same parallelization strategies it employs for simulating models. This approach is advantageous as it removes the need to transfer the model state from the CPU to the GPU and allows the GPU to be used to accelerate potentially costly initialization operations such as sampling random numbers.

Once network models have been defined using the C++ interface, GeNN will generate a *neuron* CUDA kernel for updating the neuronal state, a *synapse* kernel for simulating the propagation of spikes through synaptic connections and, for models with synaptic plasticity, a *postsynaptic learning* kernel. GeNN also generates functions for allocating memory (allocateMem), launching the initialization kernel (initialize) and launching each simulation kernel required to advance the simulation state (stepTimeGPU). The generated code can then be linked against a simulation loop provided by the user:


#**include** “model_CODE/definitions.h‭

**int** main()
{
   allocateMem();
   initialize();
   **while**(t < 100.0f) {
       stepTimeGPU();
   }
   **return** 0;
}


While this approach allows a lot of flexibility and means that visualization tools and closed-loop robotics can be tightly coupled to GeNN simulations, when combined with the use of C++ for model definition, this does make using GeNN a somewhat daunting prospect for users more used to Python-based simulators such as Brian (Stimberg et al., 2014) or PyNN (Davison et al., 2008) or graphical tools such as SpineCreator (Cope et al., 2017). For these users, GeNN can be used as a backend for other simulators. Brian2GeNN (Stimberg et al., 2018) allows models defined in Brian 2 to be translated, using code generation, into a valid GeNN simulation. Using Brian 2's backend device design, using GeNN through Brian2GeNN is as simple as issuing the command set_device(“brian2genn”) within a standard Brian 2 script. A similar interface exist for SpineCreator and an interface to PyNN (Davison et al., 2008) is currently under development.

### 2.3. Cortical Microcircuit Model

**Figure 2:**
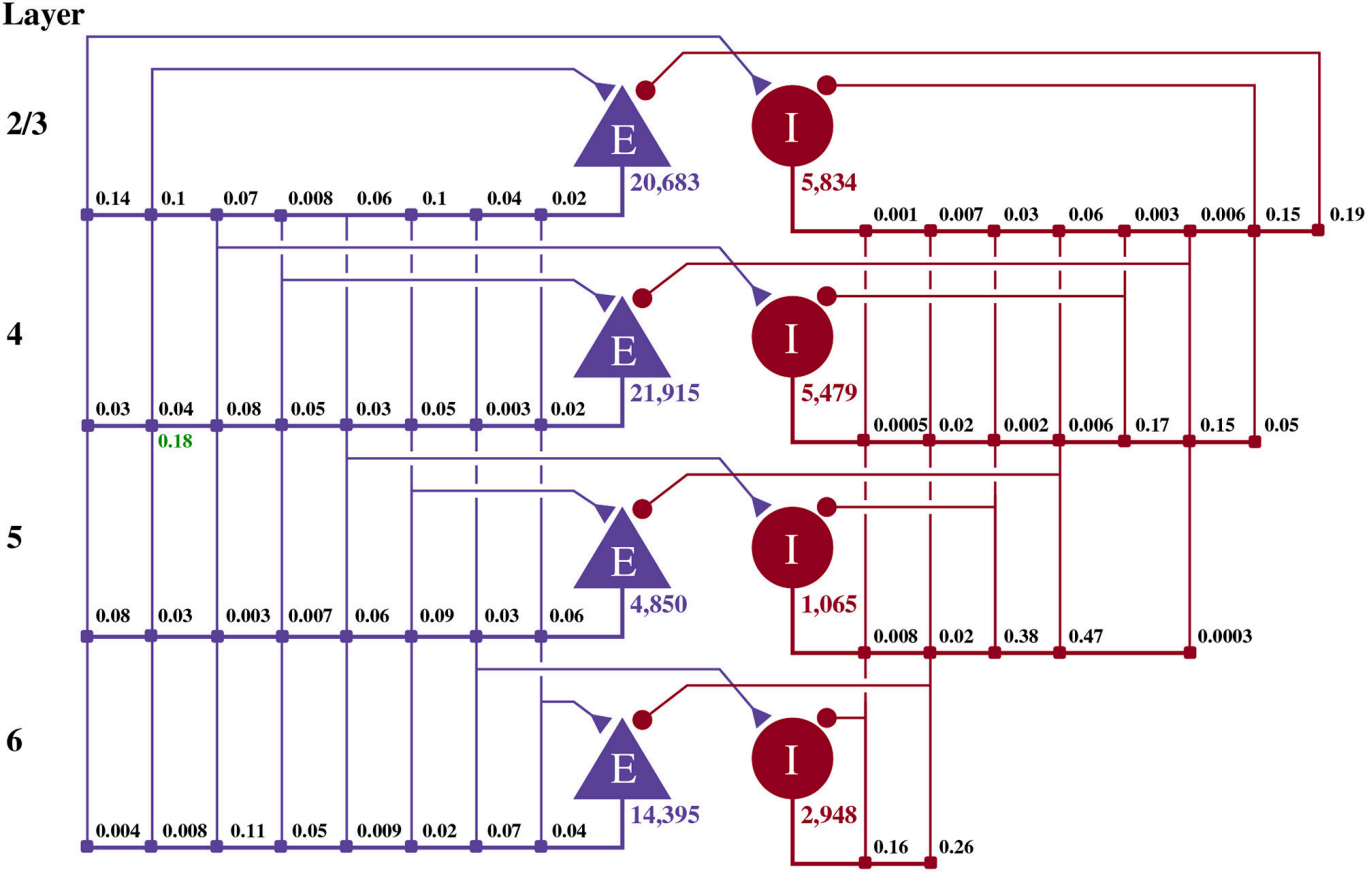
Illustration of the microcircuit model. Blue triangles represent excitatory populations, red circles represent inhibitory populations and the numbers beneath each symbol shows the number of neurons in each population. Connection probabilities are shown in small bold numbers at the appropriate point in the connection matrix. All excitatory synaptic weights are normally distributed with a mean of 0.0878 nA (unless otherwise indicated in green) and a standard deviation of 0.008 78 nA. All inhibitory synaptic weights are normally distributed with a mean of 0.3512 nA and a standard deviation of 0.035 12 nA.

This model of 1 mm3 of early-sensory cortex was developed by Potjans and Diesmann (2014) and consists of 77,169 neurons, divided into separate populations representing cortical layers 2/3, 4, 5, and 6. Each layer is modeled by an excitatory and an inhibitory neuron population as shown in Figure 2. Neurons in each population are connected randomly with population-specific densities derived from an extensive review of the anatomical literature resulting in a total of approximately 0.3·109 synapses. Beside this structured connectivity, all synaptic strengths and transmission delays are normally distributed. The membrane voltage (*V*_*j*_) of each neuron is modeled as a leaky integrate-and-fire (LIF) unit:

(1) $$\begin{array}{l} \tau_{m}\frac{dV_{j}}{dt}=\left(V_{j}-V_{rest}\right)+R_{m}I_{{in}_{j}} \end{array}$$

where τ_*m*_ and *R*_*m*_ represent the time constant and resistance of the neuron's cell membrane, *V*_*rest*_ defines the membrane voltage the neuron returns to if it receives no synaptic input and *I*_*in*_*j*__ represents the input current to the neuron. When the membrane voltage crosses a threshold (*V*_*thresh*_) a spike is emitted, the membrane voltage is reset back to *V*_*rest*_ and a countdown timer is started which, while running, disables the integration of further input thus providing a simulated refractory period. Incoming spikes induce an exponentially-shaped input current in *I*_*in*_*j*__:

(2) $$\begin{array}{l} \tau_{syn}\frac{dI_{{in}_{j}}}{dt}=-I_{{in}_{j}}+I_{p_{j}}+\overset{n}{\underset{i=0}{\sum }}w_{ij}\underset{t_{i}^{f}}{\sum }\delta \left(t-t_{i}^{f}\right) \end{array}$$

where τ_*syn*_ represents the time constant with which any spikes (modeled as Dirac delta functions δ) from *n* presynaptic input neurons occuring at time *t* are integrated. In addition to its synaptic input, each neuron in the network also receives an independent Poisson input current *I*_*p*_*j*__ (also exponentially shaped by Equation 2) which represents input from adjacent cortical regions. Finally, *w*_*ij*_ represents the peak synaptic input current of the synapse between the presynaptic neuron *i* and the postsynaptic neuron *j*. For a full description of the model parameters please refer to Potjans and Diesmann (2014, Tables 4, 5). In the remainder of this section we will concentrate on describing the strategies used to parallelize the initialization and subsequent simulation of this network.

Although the equations describing the neuron dynamics (Equations 1, 2) are coupled, in our GeNN model, the continuous terms of the two equations are solved separately so that the synaptic input current *I*_*in*_*j*__ entering into Equation (1) is effectively treated as a constant during each simulation timestep. As Rotter and Diesmann (1999) explain, this approach leads to a delay of one simulation timestep compared to the exact solution. However, by separating the solving of these equations, populations of neurons whose input synapse have different dynamics can be trivially supported. For example, while a single exponential may be a good approximation of some inhibitory synapses, for other types of synapse the rise time of the post synaptic potential may be vital (Van Vreeswijk et al., 1994). Additionally, from a software engineering point-of-view, separating the solving of these equations allows for better encapsulation of neurons and synapses.

Simulating a homogeneous *population* of neurons is an ideal task for a SIMD or SIMT device such as a GPU: the neurons do not communicate with each other within a timestep and, aside from the relatively rare times that they spike, each neuron will be simulated using exactly the same code path. Therefore, neural simulation can be trivially parallelized by simulating each neuron on a single thread that fetches the neuron's state variables from global memory into registers at the start of each timestep, advances the simulation state and writes back the state variables. As long as the state variables are laid out correctly in memory, the required memory operations can be coalesced so that a 4 B state variable can be read for 32 neurons in a single 128 B transaction—the most efficient way to access the global memory. The Poisson input current (*I*_*p*_*j*__) is calculated by generating a Poisson deviate every simulation timestep, using the technique described by Devroye (2013, p504), and multiplying this by a population-specific weight. When a neuron's spiking threshold condition is met, the thread simulating the neuron writes the index of the neuron within the population to a shared memory array. After all the neurons in a population have been updated, the shared memory arrays containing the indices of the neurons in each thread block which spiked are combined into a global memory array—forming a record of all the neurons in the population which have spiked in the current simulation timestep.

**Figure 3:**
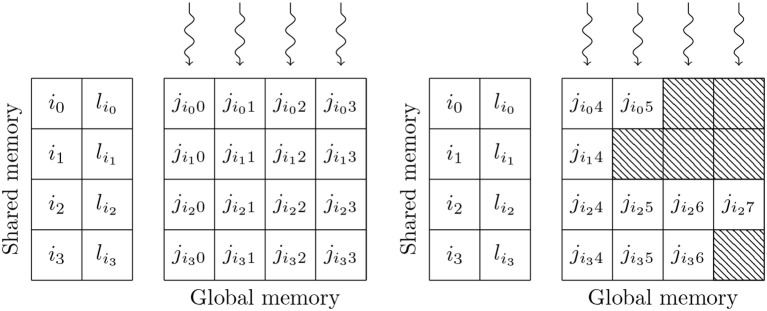
GPU parallelization of sparse synaptic matrix processing across two thread blocks each with 4 threads. *i*_0_, …, *i*_3_ contain the indices of presynaptic spikes. *l*_*i*_0__, …, *l*_*i*_3__ contain the lengths of the corresponding matrix rows. *j* contains the indices of the postsynaptic target neurons. Snaking lines indicate CUDA threads. Hatching indicates padding entries.

Simulating the spikes propagating between two populations of neurons through sparsely connected synapses is, at first glance, less suitable for GPU parallelism. However, on modern GPU hardware, this can also be implemented in an efficient manner using the data structures shown in Figure 3. These structures consist of multiple 2D arrays with rows representing the synapses coming from individual presynaptic neurons and with enough columns to contain the largest number of postsynaptic targets any presynaptic neuron connects to. One of these 2D arrays contains the indices of the postsynaptic neurons (*j*) and additional arrays are allocated for any individual synaptic state variables such as the synaptic weight (*w*_*ij*_) or dendritic delay (*d*_*ij*_). In order to simulate dendritic delays, GeNN also allocates a delay *ring-buffer* between each pair of connected populations consisting of a *D*_*max*_×*N*_*post*_ 2D array where *D*_*max*_ is the maximum dendritic delay and *N*_*post*_ is the number of postsynaptic neurons. Each block of *N*_*block*_ CUDA threads (in Figure 3 *N*_*block*_ = 4) is responsible for processing *N*_*block*_ columns of the matrix. Processing begins by using the *N*_*block*_ threads to fetch the indices of *N*_*block*_ presynaptic spikes written to global memory by the presynaptic population's neuron kernel (*i*_0_, …, *i*_*N*_*block*_−1_) and the lengths of the corresponding rows of the matrix (*l*_*i*_0__, …, *l*_*i*_*N*_*block*_−1__) into shared memory (so that these will be accessable to all threads in the block during the next phase). Threads are then synchronized and loop through the *N*_*block*_ rows with each thread processing the synapse in their column. In the case of the simple static synapses described by Equation (2), this processing simply consists of reading the index of the postsynaptic target neuron along with the weight *w*_*ij*_ and delay *d*_*ij*_ associated with the connection and using an atomic add operation to add the weight to row (*i*+*d*_*ij*_) mod *D*_*max*_ of the dendritic delay ring-buffer. Postsynaptic neurons then read their synaptic input (the $\overset{n}{\underset{i=0}{\sum }}w_{ij}\underset{t_{i}^{f}}{\sum }\delta \left(t-t_{i}^{f}\right)$ term in Equation 2) from row *i* mod *D*_*max*_ of the dendritic delay ring buffer.

This process is repeated until all incoming spikes are processed. While this parallelism strategy may seem counter-intuitive, it typically performs much better than the naïve approach of using one thread per incoming spike as it not only exposes much more parallelism, but also results in perfectly coalesced memory read operations. For example, in a simulation with a 0.1 ms timestep, a population of 10,000 neurons firing at an average rate of 10 Hz will only, on average, emit 10 spikes in a single timestep. However, if this population is connected to another population of same size with a 10 % connection probability, the connection matrix will have over 1,000 columns resulting in 2 orders of magnitude more parallelism being exposed. Using the data structures described in this section, a GeNN simulation of the cortical microcircuit model requires 3.1 GB of device memory.

An additional advantage of the data structure shown in Figure 3 is that, as long as we know the *maximum* length of any row, memory can be allocated by the host without having to perform any further calculations, meaning that the connectivity itself can be initialized on the GPU. In this model the density of the synaptic connections between a pair of neuronal populations is specified in terms of a total number of random synapses (*N*_syn_) (a FixedNumberTotal connector in PyNN). The maximum row length when connecting a presynaptic population with *N*_pre_ neurons to a postsynaptic population with *N*_post_ neurons using this connectivity can be obtained by evaluating the inverse cumulative distribution function (CDF) of $\text{Binom}\left[N_{\text{syn}},\frac{N_{\text{post}}}{N_{\text{post}}*N_{\text{pre}}}\right]$ with a suitably high probability (we use $P=0.9999^{\frac{1}{N_{\text{pre}}}}$. Once memory is allocated for the data structure, the first stage in initializing the connectivity is to determine how many of the total synapses *N*_syn_ end up in each row by sampling from the multinomial distribution Mult[*N*_pre_ * *N*_post_, {*P*_*row*_, *P*_*row*_, …, *P*_*row*_}] where $P_{row}=\frac{N_{\text{post}}}{N_{\text{syn}}}$. This operation cannot be efficiently parallelized so must be performed on the host but, once the length of each row is determined, the postsynaptic targets of the synapses can be initialized in parallel by sampling from the discrete uniform distribution Unif[0, *N*_post_] using *N*_pre_ CUDA threads. While this works mathematically, in order to improve the locality of memory accesses, synapses should be sorted into ascending order. This would be trivial to implement in CPU code but, without enough shared memory for each CUDA thread to store a copy of its corresponding row, an in-place sort in global memory would be very slow. It would be possible to use a more complex parallel sorting algorithm such as that proposed by Awan and Saeed (2016) but, as GPU architectures typically have very high floating point maths throughput, we instead take an alternative approach. Rather than sampling directly from Unif[0, *N*_post_] we sample from its 1st order statistic – Beta[1, *N*_post_] – essentially the next smallest value. In general, the Beta distribution cannot be sampled from in constant time. However, if *X* ~ Beta[1, *N*_post_], 1 − *X* ~ Beta[*N*_post_, 1] and therefore −*ln*(1 − *X*) ~ Exponential[*N*_post_] – a much simpler problem as the exponential distribution can be sampled in constant time using the inversion method (Devroye, 2013, p. 29).

### 2.4. Balanced Random Network With Spike-Timing Dependent Plasticity

**Figure 4:**
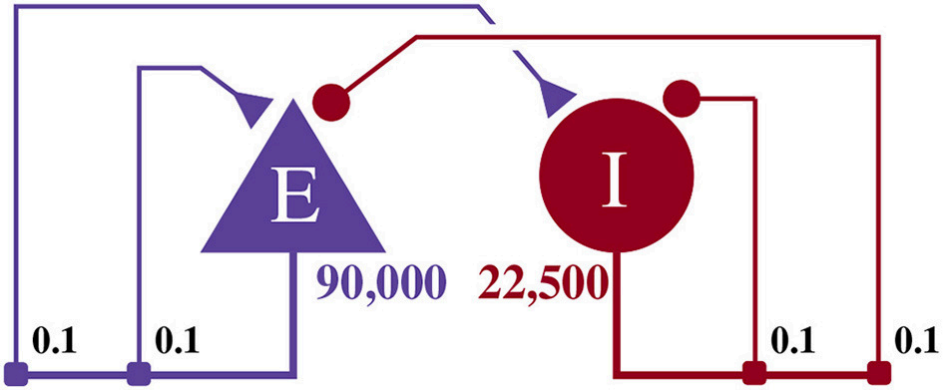
Illustration of the balanced random network model. The blue triangle represents the excitatory population, the red circle represents the inhibitory population, and the numbers beneath each symbol show the number of neurons in each population. Connection probabilities are shown in small bold numbers at the appropriate point in the connection matrix. All excitatory synaptic weights are initialized to 0.045 61 nA and all inhibitory synaptic weights are initialized to 0.228 05 nA.

This model, as illustrated in Figure 4, consists of an exitatory neuron population with 90,000 excitatory neurons and an inhibitory population containing 22,500 inhibitory neurons. This scale is necessary to achieve a realistic number of incoming connections per neuron of ≈10, 000 (Braitenberg and Schüz, 2013) with a biologically plausible connection probability of ≈0.1.

Similar to the microcircuit model described in the previous section, this model uses LIF neurons with current inputs. However, rather than filtering the input current (*I*_*in*_*j*__) using a single exponential, this model uses slightly more complex *alpha* synapses (Rall, 1967) which provide a closer match to the dynamics of biological synapses,

(3) $$\begin{array}{l} \tau_{syn}\frac{dI_{{in}_{j}}}{dt}=x_{j}-I_{{in}_{j}} \end{array}$$

(4) $$\begin{array}{l} \tau_{syn}\frac{dx_{j}}{dt}=-x_{j}+I_{p_{j}}+\overset{n}{\underset{i=0}{\sum }}w_{ij}\underset{t_{i}^{f}}{\sum }\delta \left(t-t_{i}^{f}\right) \end{array}$$

where *x*_*j*_ represents a second state variable and all other terms maintain the same meanings they had in Equation (2). Nonetheless, Equations (3, 4) have trivial algebraic solutions meaning they can be simulated using the same scheme described in the previous section.

The synapses in this model are plastic, i.e., the weights *w*_*ij*_ are changing over time according to an STDP rule. Even leaving aside synaptic plasticity rules which use postsynaptic membrane voltage (Brader et al., 2007; Clopath et al., 2010) rather than postsynaptic spike times or include “third factors” such as dopamine (Izhikevich, 2007), there is a plethora of different STDP formalizations (see Morrison et al. (2008) for a review). For the model described in this section, Morrison et al. (2007) chose to use a rule that modifies the synaptic weight (*w*_*ij*_) between a pre and postsynaptic neuron based solely on the relative timing of pre (*t*_pre_) and postsynaptic (*t*_post_) spikes (Δ*t* = *t*_post_ − *t*_pre_):

(5) $$\begin{array}{l} \Delta w_{ij}=\;\{\begin{array}{ll} \lambda w_{0}^{1-\mu }w_{ij}^{\mu }e^{-\frac{\left|\Delta t\right|}{\tau }} & if\Delta t>0\\ -\lambda \alpha w_{ij}e^{-\frac{\left|\Delta t\right|}{\tau }} & if\Delta t\leq 0 \end{array} \end{array}$$

where λ represents the learning rate, *w*_0_ defines a reference weight and μ allows the potentiation term to be set as entirely multiplicative (μ = 1), entirely additive (μ = 0) or somewhere in between. As discussed by Morrison et al. (2007), in the model presented in this section, μ is set to 0.4 so as to match the data recorded by Bi and Poo (1998). Finally τ defines the time constant of the STDP kernel and α controls the relative strength of potentiation and depression. Morrison et al. use this rule with an *all-to-all* spike-pairing scheme meaning that each of the pairs formed by a presynaptic spike and all preceding postsynaptic spikes (and vice-versa) should be considered. For the full description of the model parameters, please refer to Morrison et al. (2007, sections 3 and 4.1). In the remainder of this section we will concentrate on describing the additional steps required to parallelize models with synaptic plasticity using GeNN.

In order to implement the all-to-all spike pairing required for the model, rather than repeatedly evaluating Equation (5), we calculate updates based on per-neuron *spike traces* (Song et al., 2000; Morrison et al., 2007) with the following dynamics:

(6) $$\begin{array}{l} \frac{ds_{i}}{dt}=-\frac{s_{i}}{\tau }+\underset{t_{i}^{f}}{\sum }\delta \left(t-t_{i}^{f}\right) \end{array}$$

The value of these traces can be thought of as representing the sums of the exponential terms from Equation (5) if they were calculated for every pair of spikes. Therefore, the potentiation ($\Delta w_{ij}^{+}$) induced by the spike pairs formed by a postsynaptic spike occuring at $t_{j}^{f}$ and all preceding presynaptic spikes can be calculated using the following single update:

(7) $$\begin{array}{l} \Delta w_{ij}^{+}\left(t_{j}^{f}\right)=\lambda w_{0}^{1-\mu }w_{ij}^{\mu }s_{i}\left(t_{j}^{f}\right) \end{array}$$

Similarily, the depression ($\Delta w_{ij}^{-}$) induced by the spike pairs formed by a presynaptic spike occurring at $t_{i}^{f}$ and all preceding postsynaptic spikes can be calculated using the following single update:

(8) $$\begin{array}{l} \Delta w_{ij}^{-}\left(t_{i}^{f}\right)=-\lambda \alpha w_{ij}s_{j}\left(t_{i}^{f}\right) \end{array}$$

In GeNN, if a neuron has fired in the current timestep, its trace variables are updated in the neuron kernel by evaluating Equation (6). Synaptic depression is calculated by applying Equation (8) to each synaptic weight processed in the synapse kernel described in the previous section. Similarly, calculating synaptic potentiation involves applying Equation (7) to each synapse targetting a spiking postsynaptic neuron. However this is tricky as while the data structure shown in Figure 3 supports efficient *row-wise* access to the synapses associated with a presynaptic neuron, like many sparse matrix data structures, it does not support efficient *column-wise* accesses to the synapses associated with a postsynaptic neuron. This is a problem shared by all SNN simulators that support STDP (Brette and Goodman, 2012). Some small-scale neuromorphic systems have solved this problem in hardware using custom SRAM memories which allow both column and row-wise accesses (Seo et al., 2011). However, custom SRAMs are expensive in terms of silicon area, so many neuromorphic systems avoid the problem entirely by implementing synaptic plasticity rules which use the membrane voltage of the postsynaptic neuron rather than its spike times – meaning that no updates triggered by postsynaptic spikes are required (Qiao et al., 2015; Frenkel et al., 2018). Intel's Loihi system (Davies et al., 2018) and the SpiNNaker software developed by Galluppi et al. (2014) take an alternative approach and defer all STDP updates until the end of a “learning epoch” after which time they are processed sequentially row by row. NEST (Morrison et al., 2007) and the more recent SpiNNaker software (Knight et al., 2016) both buffer postsynaptic spikes until the next presynaptic spike occurs—allowing weight updates triggered by pre and postsynaptic spikes to be applied in order without having to make any column-wise accesses to the synaptic matrix. However, buffering postsynaptic spikes makes access to other postsynaptic variables difficult as they would also need to be buffered for an unbounded length of time until the next presynaptic spike occurs.

Deferring STDP updates ideally requires a dynamic memory structure to store postsynaptic events, which, when combined with the need to search through this data structure for events to apply, means that this approach does not appear to be well-suited for GPU implementation. Furthermore, GeNN aims to support a wide range of synaptic plasticity rules with full access to pre and postsynaptic neuron variables. Therefore, GeNN builds an additional column-major sparse matrix using the same data structure as shown in Figure 3, containing indices into the original row-wise arrays containing synaptic weights. This has the downside of doubling the memory requirements of connections when STDP is required and, as Yavuz et al. (2016) demonstrated, the resultant non-coalesced accesses to the synaptic matrix reduce performance on lower-end GPUs. However, the approaches involving buffering of events and variables described above come with their own challenges in terms of memory management and minimizing the divergence of execution between CUDA threads. Furthermore, the performance reductions due to non-coalesced memory accesses are much less severe on modern GPUs due to the ever-increasing size of their L2 cache.

**Figure 5:**
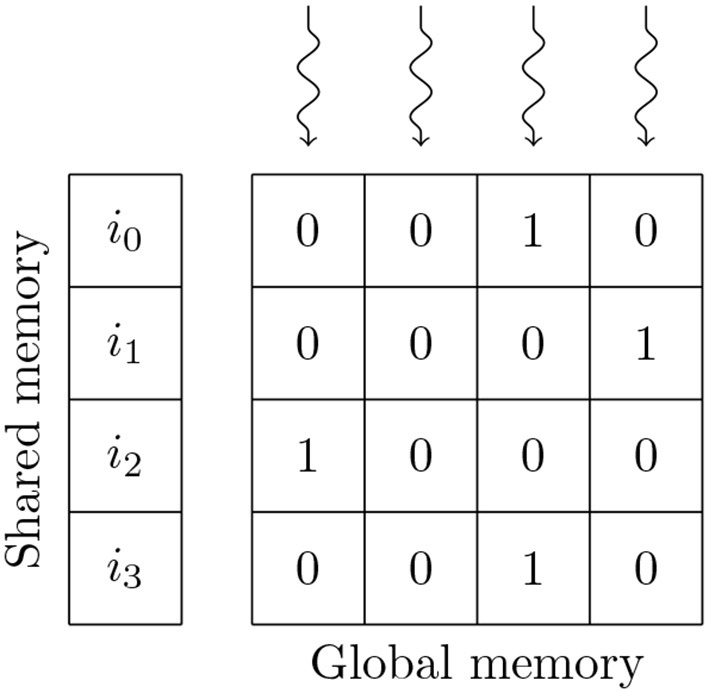
GPU parallelization of the processing of 4 postsynaptic neurons' synaptic input using a bitmask synaptic matrix and one thread blocks with 4 threads. *i*_0_, …, *i*_3_ contain the indices of presynaptic spikes. Snaking lines indicate CUDA threads. 0s and 1s indicate individual bitmask bits.

In the balanced random network model, the synaptic weights of the non-plastic connections are initialized to a constant value so the GeNN code generator can compile these constants directly into the synapse kernels. While this results in significant memory savings, it is not enough to fit the model onto GPUs with 12 GB of memory using either of GeNN's standard sparse matrix formats. We, therefore, use the alternative *bitmask* data structure shown in Figure 5 to store the non-plastic connections on these GPUs. When using the *bitmask* data structure, the connections between a presynaptic population with *N*_pre_ neurons and a postsynaptic population with *N*_post_ neurons are stored using a *N*_pre_×*N*_post_ bit bitfield (rounded up to the nearest 32 bit word). For example, the connections between the excitatory (90,000 neurons) and inhibitory populations (22,500 neurons) in the balanced random network model can be stored in 241 MiB using a bitmask rather than 867 MiB when using the data structure described in the previous section. Using the bitmask approach reduces the total amount of device memory required to simulate this model in GeNN from 11.5 to 10.2 GB. The bitmask data structure is processed using a CUDA thread to accumulate each postsynaptic neuron's input into a register every simulation timestep. Each of these threads loops through the incoming spikes stored in the shared memory data structure described in the previous section and, if the corresponding bit in the bitmask is set, adds the synaptic weight to the register.

Similarly to the data structure shown in Figure 3, the amount of memory required to store synapses in the bitmask data structure can be be calculated without any knowledge of the connectivity within, meaning that synapses stored in this format can also be initialized on the GPU. In this model, the density of the synaptic connections is described using a probability of connection *P* (a FixedProbabilityConnector connector in PyNN). Therefore, whether a synapse exists between a pair of pre and postsynaptic neurons can be described using a Bernoulli distribution Bern[*P*_conn_]. While the Bernoulli distribution can be sampled by repeatedly drawing from the uniform distribution Unif[0, 1] and comparing each sample to *P*, this is innefficient for sparse connectivity. Instead we sample from the geometric distribution Geom[*P*_conn_] which describes how the number of Bernoulli trials required to get a success (i.e., a synapse) is distributed. The geometric distribution can be sampled in constant time by inverting the cumulative density function (CDF) of the equivalent continuous distribution (the exponential distribution) to obtain $\frac{log\left(\text{Unif}\left[0,1\right]\right)}{log\left(1-P_{\text{conn}}\right)}$ (Devroye, 2013, p. 499). Using this approach, generating fixed probability connectivity can be performed entirely in parallel by initializing each row of connectivity using an independent CUDA thread.

## 3. Results

We implemented and tested two established computational neuroscience models. The first model is a model of a cortical microcircuit developed by Potjans and Diesmann (2014). It consists of eight populations of neurons, representing the excitatory and inhibitory populations of neurons in cortical layers 2/3, 4, 5, and 6 of a micro-column. Neurons are connected with random connectivity of densities that follow experimental observations. The model has been shown to reproduce firing characteristics observed in the cortex (Potjans and Diesmann, 2014).

The second model is a balanced random network with spike-timing dependent plasticity (Morrison et al., 2007). Synaptic plasticity is a family of mechanisms responsible for changing the strength of synaptic connections in response to neural activity and has been shown to be fundamental to biological learning (Nabavi et al., 2014). In particular, Spike Timing Dependent Plasticity (STDP) (Markram, 1997; Bi and Poo, 1998) is a popular theory which postulates that these changes are driven by the difference in timing between presynaptic spikes arriving at a synapse and the times at which the postsynaptic neuron itself spikes. In excitatory cortical (Markram, 1997) and Hippocampal (Bi and Poo, 1998) neurons, synapses at which a presynaptic spike is closely followed by a postsynaptic spike are strengthened, whereas those at which a postsynaptic spike precedes a presynaptic spike are weakened, so introducing a causal learning rule. Adding STDP to SNN simulations, however, typically increases the computational cost of simulating them significantly. Morrison et al. (2007) reported that adding plasticity to their simulations slowed them down by “a factor of less than 10” and Knight and Furber (2016) found that, in the **best** case, simple STDP plasticity reduced the performance of the SpiNNaker neuromorphic system by approximately 6 ×. Furthermore, the dynamics of neural systems with plasticity operating on biologically-plausible time scales take several orders of magnitude more time to stabilize meaning that longer simulations are required and, as Morrison et al. argue, it is vital to perform experiments on STDP in models with full-scale connectivity to avoid synchronization artifacts.

Balanced random networks such as this have been shown to reproduce some of the dynamics seen in the neocortex (Brunel and Hakim, 1999; Brunel, 2000). Morrison et al. showed that adding STDP to their model did not disrupt its dynamics and, as long as a suitable STDP rule is used, the synaptic weights will settle into a stable unimodal distribution.

### 3.1. Correctness

In this section we will focus on confirming the correctness of our simulations of the microcircuit model (Potjans and Diesmann, 2014) described in section 2.3 using the methodology described by van Albada et al. (2018). Additionally we will compare the results of simulations of the balanced random network model described in section 2.4 to those reported by Morrison et al. (2007).

#### 3.1.1. Cortical Microcircuit Model

van Albada et al. (2018) performed an in-depth analysis of the correctness of simulations of the microcircuit model—running both on NEST and on the SpiNNaker neuromorphic system—using NEST running in “precise” mode as a ground-truth. In “precise” mode, rather than constraining spike events to simulation time steps, NEST communicates the exact time at which neurons' membrane voltages cross the threshold between the nodes simulating the model (Hanuschkin et al., 2010).

In order to assess correctness, we simulated 10 s biological time of the model. As van Albada et al. describe, the first 1 s of spike data from each 10 s simulation was discarded in order to remove any transients. We then calculated the average firing rates and the covariance of interspike intervals (CV ISI) for each neuron in the model over the remaining 9 s of the simulation using the Elephant (Yegenoglu et al., 2018) package. We also picked 200 (this was a trade-off between accuracy and analysis time chosen by van Albada et al.) active neurons from each population, binned their spike trains into 2 ms bins (corresponding to the refractory time of the neurons) and calculated the Pearson correlation coefficient matrix between each disjoint pair of neurons.

The same measures were calculated for GeNN and for a NEST simulation run in “precise” mode and histograms of all three measures were produced for both simulations using bins calculated from the NEST data using the Freedman-Diaconis rule (Freedman and Diaconis, 1981). The histograms were smoothed with Gaussian kernel density estimation performed using the scipy.stats.gaussian_kde function with bandwidths of 0.3 s-1, 0.04 and 0.002 for the average firing rates, CV ISI and correlation respectively.

**Figure 6:**
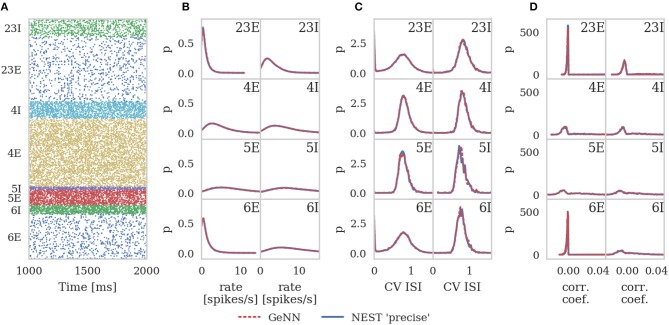
Spiking output of cortical microcircuit model with Poisson input. All measures are calculated over the last 9 s of the simulation and histogram bin widths are determined using the Freedman-Diaconis rule. **(A)** Raster plot showing spike times (dots) of neurons from each population. The spikes of 5% of neurons (vertical) are shown. **(B)** Single-neuron firing rates of all neurons. **(C)** CV ISI, a measure of irregularity of all neurons. **(D)** Correlation coefficients between binned spike trains for 200 neurons in each population.

**Figure 7:**
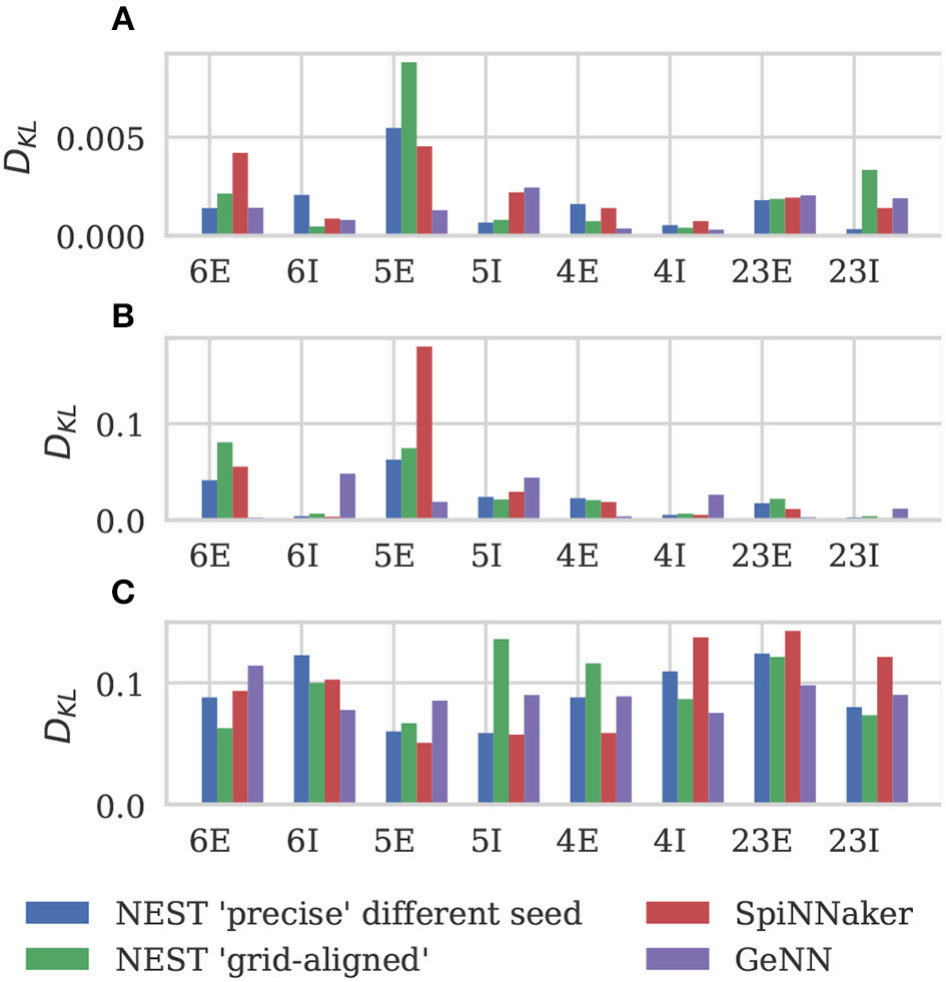
Comparison of per-population distributions of dynamical properties shown in Figure 6. Comparisons calculated using the Kullback-Leibler (KL) divergence with NEST running in “precise” mode as a reference. For comparison, KL divergences for NEST running in “grid-aligned” mode and SpiNNaker are read off figure presented by van Albada et al. **(A)** Single-neuron firing rates. **(B)** CV ISI. **(C)** Correlation coefficients.

Figure 6 shows the results of this analysis. Visually it is clear that the per-population distributions are highly similar and, to quantify this, we calculated the Kullback-Leibler (KL) divergences using the “precise” NEST data as the reference. Figure 7 shows the KL divergences calculated from our GeNN simulation as well as those reported by van Albada et al. (2018) for their grid-aligned NEST and SpiNNaker simulations and between two “precise” NEST simulations with different random number generator seeds. Similarly to those calculated from the SpiNNaker and grid-aligned NEST simulations, the KL divergences from our GeNN simulation are comparable in size to those caused by changing the random number generator seed.

#### 3.1.2. Balanced Random Network

**Figure 8:**
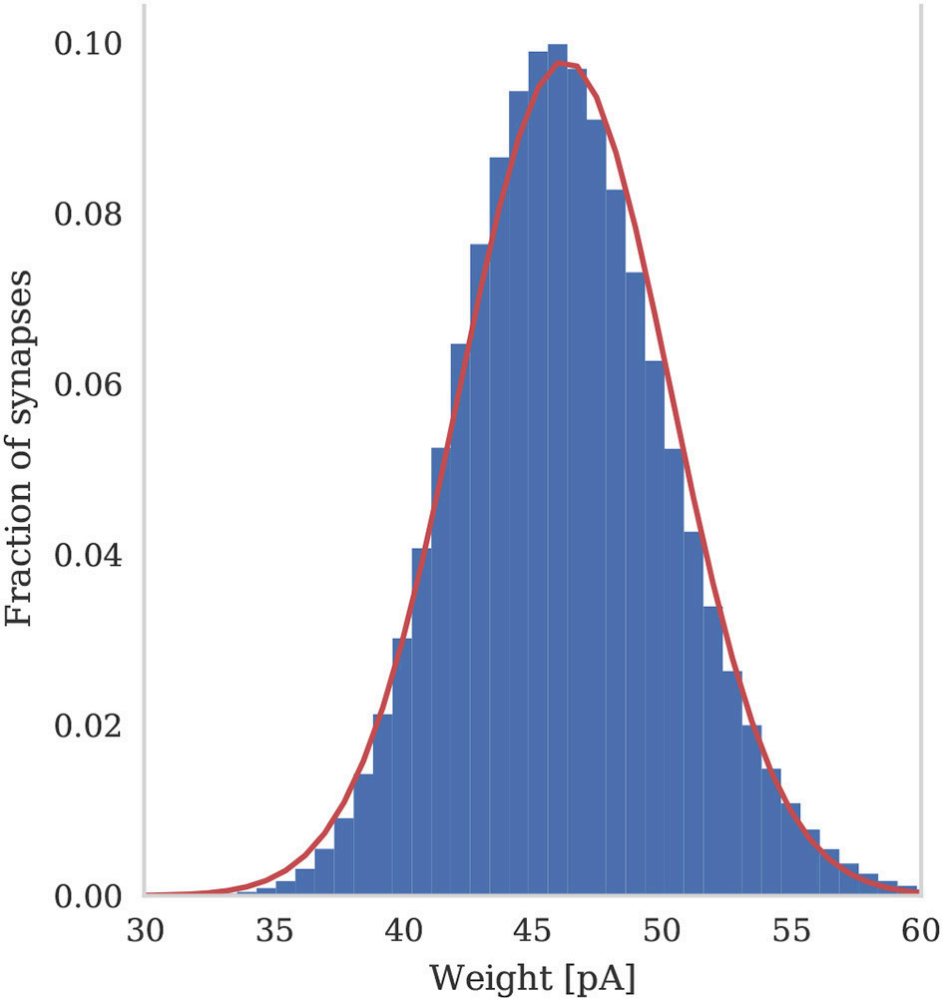
Histogram of the synaptic weight distribution obtained after 2,000s of simulation. The solid red line shows the gaussian distribution with μ_*w*_ = 46.25 and σ_*w*_ = 4.07.

To assess the correctness of our implementation of the balanced random network model described in section 2.4, we simulated the network for 2,000s of biological time and compared the final weight distribution and the statistics of the last 50 s of spiking activity with those reported by Morrison et al. (2007). The calculated statistics are listed in Table 1 and the final weight distribution is shown in Figure 8. To quantify the network dynamics resulting from these synaptic weights, we calculate the mean firing rate and CV ISI of all the excitatory neurons in the network using the Elephant (Yegenoglu et al., 2018) package. The mean firing rate and CV ISI values listed in Table 1 suggest that our model had settled into a very similar asynchronous-irregular regime to that reported by Morrison et al. (2007). Our model exhibited fast oscillations throughout the simulation and, to quantify the resultant variation in spike rate, we calculated a histogram with 3 ms bins from the output spike trains of 1,000 excitatory neurons. By dividing the variance of each bin's spike count by its mean we calculated a Fano factor which, again, was very simular to that reported by Morrison et al. (2007). As Pauli et al. (2018) thoroughly demonstrate, reproducing results from SNN models on different simulators can be difficult, especially with models of this age where the original code is not publicly available. Therefore, we believe that the remaining small differences in results are likely to be due either to numerical differences caused by single-precision floating point and our use of CUDA's approximate exponential and power functions; or to subtle differences in the order of operations between GeNN and NEST.

**Table 1:** Comparison of statistics reported by Morrison et al. (2007) with those obtained from our GeNN simulations.

**Statistic**	**Value reported by**	**Value obtained from**
	**Morrison et al. (2007)**	**GeNN simulation**
Mean weight (pA)	45.65	46.25
Weight standard deviation (pA)	3.99	4.07
Mean spike rate (Hz)	8.8	8.8
Covariance of interspike interval	0.88	0.86
Fano factor	8.5	8.3

### 3.2. Performance

To assess the performance of our GPU simulations we chose a selection of GPUs listed in Table 2—covering a range of financial and power budgets. CUDA abstracts away the degree of parallelism exposed by the application from the amount of hardware parallelism available so we can run a model that uses 80,000 threads on a GPU with many fewer CUDA cores. However, memory is a harder constraint so, while all of the GPUs listed in Table 2 can run the microcircuit model described in section 2.3, due to the increased memory requirements of STDP connections, only the two “Tesla” GPUs have enough memory to run the balanced random network model described in section 2.4.

**Table 2:** GPU devices.

**Model**	**Thermal design**	**Architecture**	**Num**.	**Memory**	**Memory**	**Max single-precision**
	**power (TDP)**		**CUDA**	**capacity**	**bandwidth**	**performance**
	**[W]**		**cores**	**[GB]**	**[GB s-1]**	**[GFLOPS]**
GeForce 1050 Ti	75	Pascal	768	4	112	2,100
Jetson TX2	15	Pascal	256	8^a^	58.4	750
Tesla K40c	235	Kepler	2,880	12	288	4,290
Tesla V100	250	Volta	5,120	16	900	14000

a *Memory is shared between CPU and GPU*.

We measured the performance of both models by querying the std::chrono::high_resolution_clock around the simulation loop to obtain a total *simulation time*. In order to analyse how time was spent in the different GPU kernels we also used CUDA's own event timing system (NVIDIA Corporation, 2018a, Section 3.2.5.6.2) to record the time taken by the neuron and synapse simulation kernels as well as the postsynaptic learning kernel in the balanced random network model. By dividing the simulation time by the length of the simulation in *biological time*, we can then obtain an estimate of the average simulation performance relative to real-time.

#### 3.2.1. Cortical Microcircuit Model

**Figure 9:**
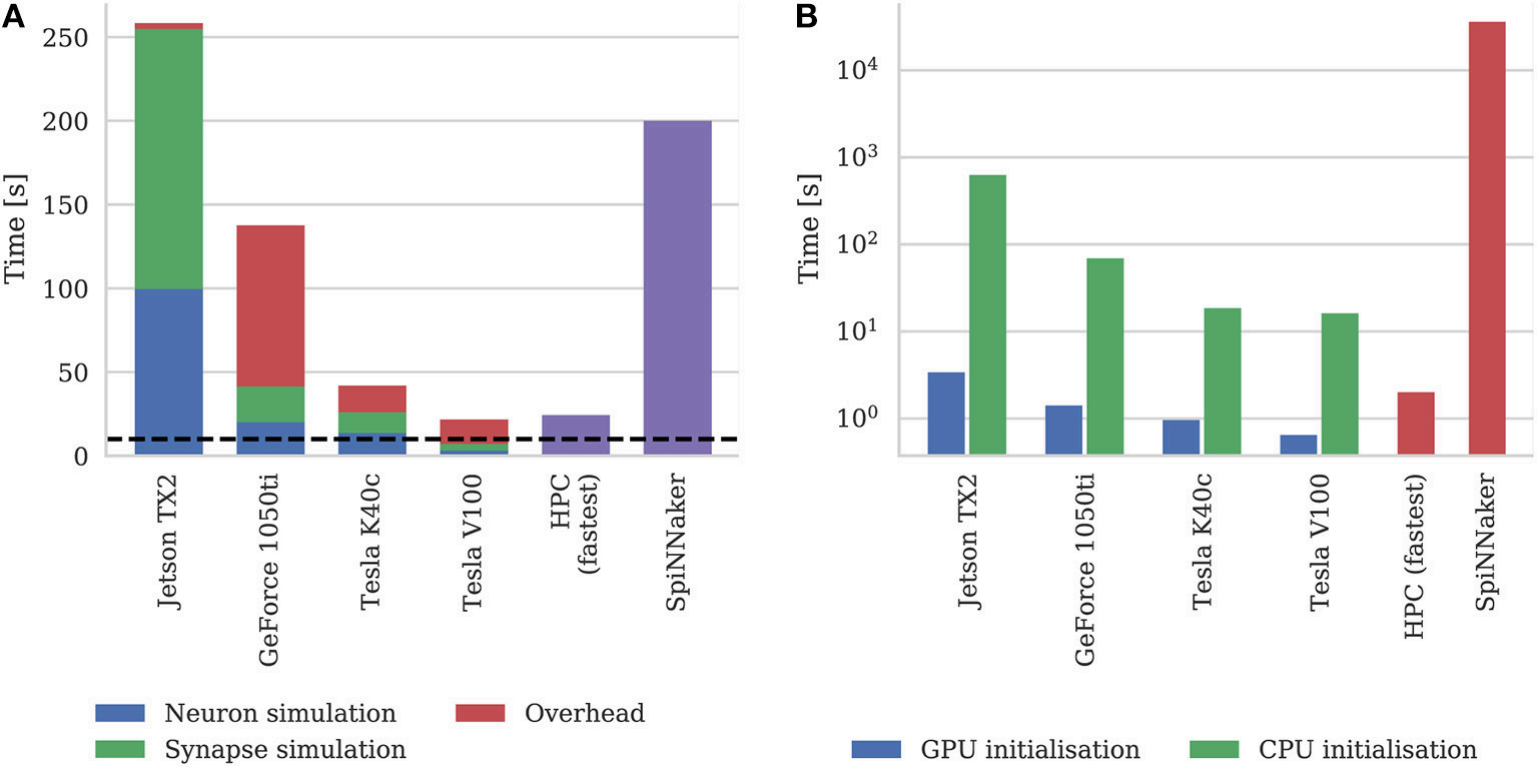
**(A)** Simulation times of the microcircuit model running on various GPU hardware for 10 s of biological time. SpiNNaker and fastest HPC simulation times (12 nodes) presented by van Albada et al. (2018) included for comparison. “Overhead” in GPU simulations refers to time spent in simulation loop but not within CUDA kernels. The dotted horizontal line indicates realtime performance. **(B)** Initialization times of the microcircuit model running on various GPU hardware. SpiNNaker and fastest HPC simulation times (32 nodes) presented by van Albada et al. (2018) included for comparison.

Figure 9A shows the simulation times of the microcircuit model running on each GPU for 10 s of biological time, including the times taken by neuron and synapse simulation kernels. Compared to the smaller point neuron benchmark presented by Yavuz et al. (2016), even though each neuron in our model receives up to 10× as many synaptic inputs, the simulation time is more evenly split between the simulation of neurons and synapses. This is partly because our simulations are running with a smaller 0.1 ms timestep meaning that less presynaptic spikes are processed each timestep. Additionally, in the newer version of GeNN used in this paper, the generation of Poisson noise takes place in the neuron kernel rather than in the separate kernel used by Yavuz et al. (2016).

In general, as one would expect, the two Tesla GPUs perform best with the newer Tesla V100 system achieving a faster simulation speed than was possible on the CPU-based HPC cluster (van Albada et al., 2018). However even the GeForce 1050ti—which is a low-end gaming GPU—can simulate the model faster than the SpiNNaker system.

As discussed in section 2.2, as well as parallelizing neuron and synapse simulation code, the latest version of GeNN also parallelizes the initialization of model state variables and connectivity using the GPU. Figure 9B shows the initialization time of the microcircuit simulation when initialization is performed on the CPU compared to the GPU. Even on the two Tesla systems which have Intel Xeon CPUs with high single-threaded performance, using the GPU for initialization, results in a speedup of around 20× and on the Jetson TX2, with its much slower ARM A57 CPU, GPU initialization is more than 150× faster.

Figure 9B also includes the initialization times for SpiNNaker and the fastest HPC configuration presented by van Albada et al. (2018) The scaling plot for HPC initialization presented by van Albada et al confirms the trivially parallelisable nature of network initialization compared to simulation – performance continued to increase up to 32 nodes rather than just 12 in the simulation. However, all three desktop GPU systems still perform network initialization in a shorter time than the HPC system. Diamond et al. (2016) concluded that initialization and loading time was a big problem for neuromorphic systems and SpiNNaker clearly still has issues in this area as initializing and loading the microcircuit network onto the SpiNNaker system takes approximately 10 h. This is approximately 50× slower than the Jetson TX2 when only one of its ARM cores is used for initialization.

**Figure 10:**
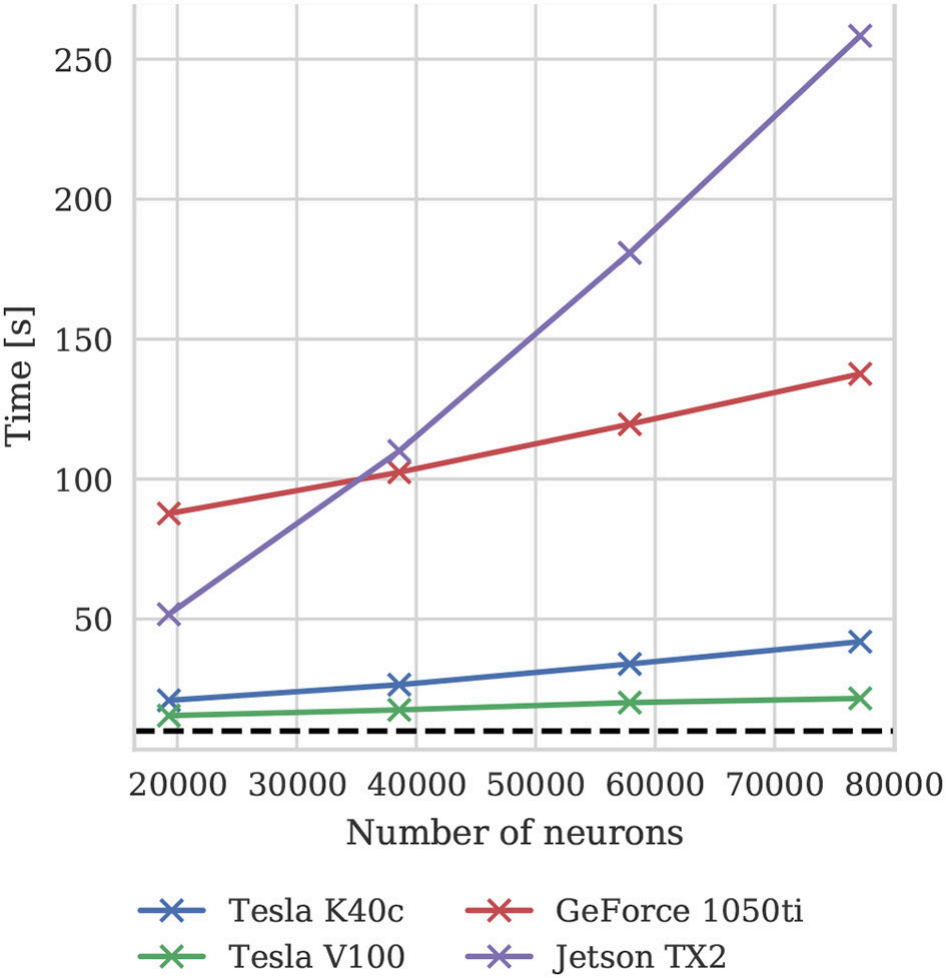
Simulation times of the microcircuit model running for 10 s of biological time at different scales on various GPU hardware. Downscaling rules described by van Albada et al. (2015) are employed to maintain spiking statistics at all scales. The dotted horizontal line indicates realtime performance.

To illustrate how the run-time of GPU simulations varies with model size, we also simulated scaled down versions of the microcircuit model on all four GPU devices. Scaling is performed by using the downscaling rules described by van Albada et al. (2015) to produce versions of the microcircuit model with a total of *N*_*t*_ neurons using a scaling factor $K=\frac{N_{t}}{77169}$. The size of each population of neurons and the total number of connections between them are scaled down by *K*. The firing rate of the Poisson background input provided to each population is also scaled down by *K* and partially replaced by a DC input current scaled by $\left(1-\sqrt{K}\right)$ (meaning that it is not present in the full-scale model). Finally the mean synaptic weights are multiplied by $\sqrt{K}$. The effect of these scaling rules is to preserve the spiking statistics at all model scales. Figure 10 shows the results of these simulations and suggests that, because at the tested scales there are always many more neurons than CUDA cores and the activity remains constant, simulation times scale approximately linearly with the number of neurons and synapses. For a more in depth analysis of the scaling properties of GeNN simulations we refer the reader to Yavuz et al. (2016) and Stimberg et al. (2018).

#### 3.2.2. Balanced Random Network

**Figure 11:**
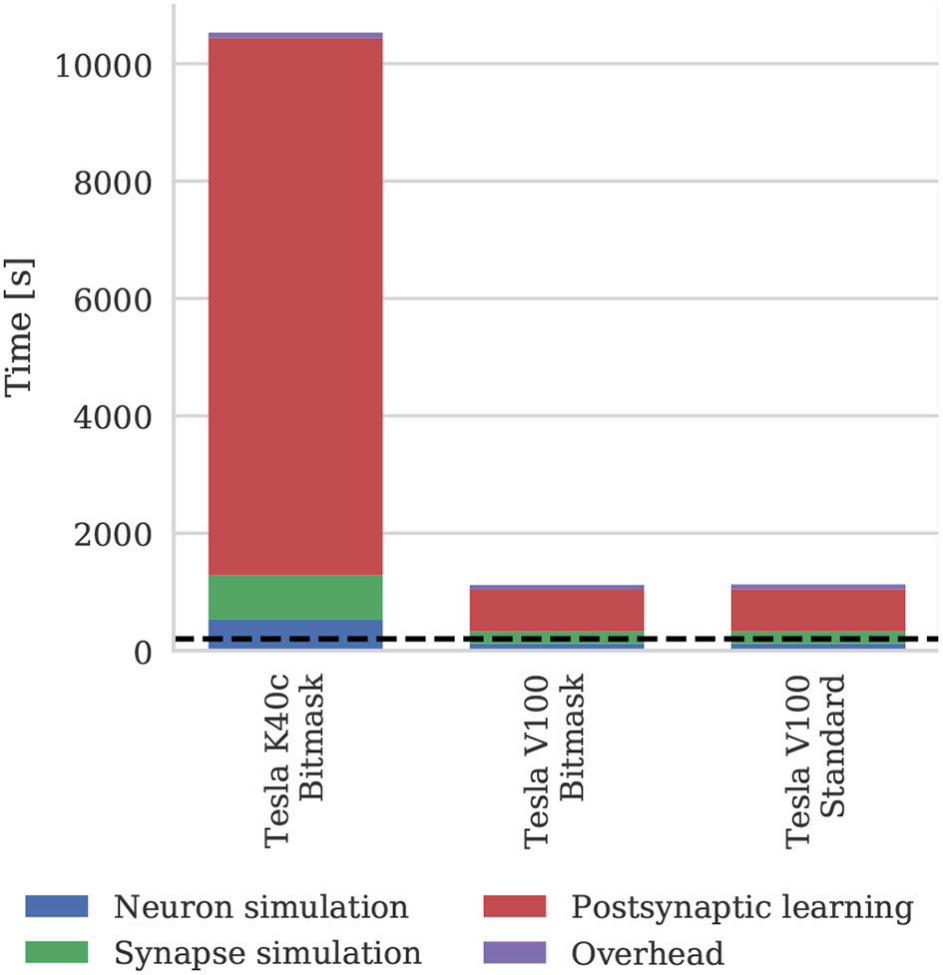
Simulation times of the balanced random network model running on various GPU hardware for 200 s of biological time. “Overhead” in GPU simulations refers to time spent in simulation loop but not within CUDA kernels. “Standard” and “Bitmask” refer to the data structure used for representing the model's non-plastic connections—the standard synaptic matrix data structure described in section 2.3 or the bitmask data structure described in section 2.4 respectively. The dotted horizontal line indicates realtime performance.

Figure 11 shows the runtime of simulations of the balanced random network model described in section 2.4. The Tesla V100 has enough memory (16 GB) to represent the model's non-plastic connections using the standard synaptic matrix data structure described in section 2.3 as well as the bitmask data structure described in section 2.4. However, Figure 11 shows that this has a negligible impact on the simulation time, suggesting that both data structures are equally efficient for large-scale models. While Morrison et al. (2007) report that their simulations of this model took 60 h to run for 1,000s of biological time on their HPC cluster—which is around 4× slower than our simulations run on the Tesla K40c—we have not included this in Figure 11 as a comparison with decade-old CPU hardware would not be a fair one.

### 3.3. Power and Energy

**Figure 12:**
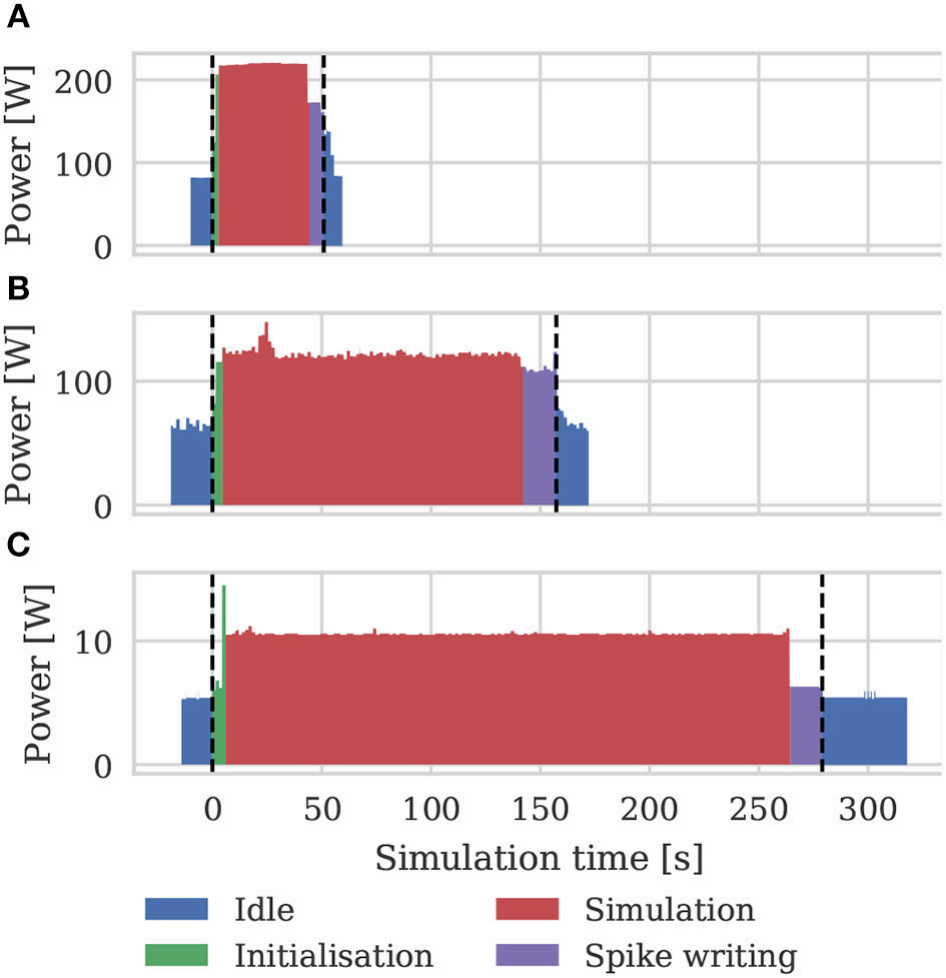
Power consumption during 10 s microcircuit simulation. Power was measured using consumer power measurement device with minimum resolution of 0.1 W at mains socket. **(A)** Tesla K40c in a workstation with a Intel Xeon E5-1620 v2 processor running Ubuntu 16.04 LTS. **(B)** GeForce 1050Ti in a desktop PC with an Intel Core i5 750 processor running Windows 7. **(C)** NVIDIA Jetson TX2 development kit running Ubuntu 16.04 LTS and JetPack 3.2.1 in maximum performance mode.

As well as recording the runtimes of the microcircuit benchmark described in the previous section, we also recorded the power usage of the systems being benchmarked using a consumer power measurement device at the mains socket. The screen of the power measurement device was recorded using a webcam, optical character recognition was performed using “Seven Segment Optical Character Recognition” developed by Auerswald and Fontana (2018) and the resultant power measurements were tagged with a time and written to disk. Figure 12 shows the power usage over time for simulations of the microcircuit model running for 10 s of biological time on each of the devices listed in Table 2 except for the Tesla V100 to which we do not have local access.

By integrating the power time series using the numpy.trapz function we calculated the energy to solution for each device as well as the energy per synaptic event—a common measure for comparing the energy efficiency of neuromorphic systems. These energy costs are listed in Table 3 alongside the energy costs presented by van Albada et al. (2018) for simulations running on SpiNNaker and a CPU-based cluster. Whilst we were unable to measure the energy of the Tesla V100 system directly, Tesla GPUs have built in power monitoring which shows that the Tesla V100 drew a maximum of 88 W compared to 107 W for the Tesla K40c. As the workstation containing the Tesla K40c drew 218 W while simulating the model, compared to an idle power draw of 84 W, we can estimate that the single CPU core being used by the simulation was drawing 27 W more than when the system was idle. Therefore we can estimate that, if a Tesla V100 was attached to the same workstation, the maximum power draw would be reduced to 199 W suggesting that, based on the reduced simulation time of 22 s, the simulation energy for such a system would be 0.0012 kW h and the energy per synaptic event would be 0.47 μJ.

**Table 3:** Energy cost of simulations.

**Model**	**Energy to solution**	**Simulation energy**	**Energy per synaptic event**
	******[kW h]******	**[kW h]**	**[μJ]**
GeForce 1050 Ti	0.0053	0.0051	2.0
Jetson TX2	0.00080	0.00078	0.30
Tesla K40c	0.0030	0.0028	1.08
SpiNNaker	–	0.017	5.9^a^
NEST (lowest energy)	–	0.012	4.4

*Energy to solution and simulation energy of GPU are calculated using the numpy.trapz function and the simulation energy is divided by the total number of synaptic events processed to obtain the energy per synaptic event. For comparison, simulation energies and energies per synaptic event for SpiNNaker and the NEST simulation with the lowest simulation energy (2 nodes) are read off the figure presented by van Albada et al. (2018)*.

a *This energy per synaptic event is calculated after the “idle” power of the SpiNNaker system has been taken into account*.

From Figure 12 we can see that even an idling workstation draws on the order of 100 W and, as van Albada et al. (2018) discuss, a single Infiniband switch has a thermal design power of over 200 W. Therefore it is somewhat unsurprising that any accelerator that allows equivalent simulations to be run on fewer nodes would significantly improve energy usage.

## 4. Discussion

### 4.1. Suitability of GPU Architectures for SNN Simulations

The 3.5× increase in peak performance and the 3.1× increase in memory bandwidth between the Tesla K40c (released in 2013) and the Tesla V100 (released in 2017) listed in Table 2 illustrate just how much GPUs have taken advantage of Moore's law scaling. This scaling is reflected in the runtime of our simulations where the cortical microcircuit model ran approximately twice as fast on the newer Tesla V100 GPU. However, the simulations of the plastic model ran more than 10× faster on the Tesla V100, suggesting that recent architectural changes have further improved the suitability of GPUs for SNN simulations. Figure 11 shows that the improved performance of the Tesla V100 is almost entirely due to the reduction of time spent in the postsynaptic learning kernel. This kernel is where synaptic potentiation is applied using the approach outlined in section 2.4 in which an additional column-major sparse matrix structure is used to select weights to update in response to postsynaptic spikes. We believe that two new features of the Volta architecture (NVIDIA Corporation, 2017) used by the V100 GPU are playing a crucial role in accelerating this kernel. Firstly, Volta GPUs have 6144 KiB of L2 cache compared to only 1536 KiB in the older Kepler architecture used by the Tesla K40c, which helps to mediate the cost of non-coalesced accesses to synaptic weights. Additionally, Volta GPUs can now simultaneously execute integer and floating point operations, meaning that the pointer arithmetic required to calculate the indices into the synaptic weight matrix can be performed simultaneously with the learning rule update itself.

In our simulations of both models, we copy all spikes from the GPU to the host computer at the end of each simulation timestep. Along with the overhead involved in launching CUDA kernels every simulation timestep, the copying of spikes accounts for the majority of the “overhead” shown in Figures 9A, 11. Furthermore, because the microcircuit model has four times as many neuronal populations as the balanced random network model, copying its spiking output requires more interactions with the GPU driver, resulting in the higher overhead seen in the simulations of this model. The overhead is particularly high on the GeForce 1050ti system which we believe is due to a combination of the slower PCI express bus in this machine (PCIe Gen 2 rather than Gen 3) and issues using CUDA on display devices under Windows. When simulating the microcircuit at smaller scales this problem is exacerbated so, at the smallest scale shown in Figure 10 (19,292 neurons), these overheads account for between 65 and 90% of the simulation time on the three desktop GPUs. However, CUDA allows for memory operations to be performed asynchronously and overlapped with computation, which should allow some of this overhead to be minimized in future versions of GeNN. Because the Jetson TX2 is a system-on-chip in which the CPU and GPU cores share the same physical memory, no copying of data over the PCI Express bus is required and the overhead of the simulations running on this system are significantly lower than on any of the discrete GPUs. In fact, when we simulated the microcircuit model at the smallest scale, Figure 10 shows that the Jetson TX2 simulations actually ran **faster** than those run on the GeForce 1050ti.

In sections 2.2, 2.3, and 2.4 we discuss how GeNN uses the GPU to parallelize network initialization and, in section 3.2.1, we show how this benefits overall simulation run-times. A similar approach could be used for the analysis of simulation results, for example to reduce the 810×10^6^ plastic synaptic weights in the balanced random network model to the histogram shown in Figure 8, **before** downloading them to the host. Because of the low-level flexible nature of GeNN, this **could** already be implemented in a CUDA kernel provided by the user. However, downloading the plastic weights of the balanced random network model from the GPU to the host computer only takes around 300 ms, making it more practical to simply write these weights to disk and analyse them offline using one of the many CPU-based analysis tools available.

Unlike the simulations typically run on CPU-based systems, the GPU simulations presented in this paper use single rather than double-precision floating point and therefore have the potential for more numerical instability. Additionally, the non-associative nature of floating point operations means that, if the results from a large number of parallel threads are summed together in a non-deterministic order, results can differ between runs due to rounding errors. Villa et al. (2009) demonstrated that the result of summing 28,000 double-precision floating point numbers across 16,000 threads of a CRAY XMT system (which, in this context, has similar properties to a GPU) varied by up to 24.64 %. However, in this experiment, more numbers were summed than would occur when using any of the parallelization schemes used in our simulations and it is unclear what absolute errors the reported relative errors correspond to. Furthermore, based on the analysis we presented in section 3.1, this potential source of error did not appear to affect our simulations suggesting that using single-precision floating point and summing inputs in a non-deterministic order has a minimal effect on the dynamics of the microcircuit model.

As we discussed in the introduction, the computational requirements of training Artificial Neural Networks (ANNs) of ever-increasing size and complexity has been a major driver in the development of GPU hardware (Schmidhuber, 2015). These applications and the growing need to deploy ready-trained ANNs to perform inference in real time on low-power “edge computing” devices mean that available memory bandwidth is beginning to limit performance. Although upcoming technologies such as third generation High Bandwidth Memory (HBM3) are likely to offer increases in memory bandwidth in the future, alternative strategies are still going to be required to better utilize current GPUs for SNN simulation as well as to increase the size of models that can be simulated using embedded GPUs such as the Jetson TX2. One solution, used successfully in ANN inference and training, has been to use lower precision 16 bit floating point and even fixed point integer representations for weights (Micikevicius et al., 2018). Using smaller data types not only saves memory and memory bandwidth but, on some newer GPU hardware including the Jetson TX2, each CUDA thread can perform four 8 bit or two 16 bit operations simultaneously – significantly increasing peak performance. While lower precision types are unlikely to provide enough numerical stability for storing neuron state variables, as discussed by van Albada et al. (2018), the 16 bit fixed-point synaptic weights used by SpiNNaker provide sufficient accuracy for the microcircuit model described in section 2.3. While GeNN does not currently support these lower-precision types, we plan on extending the algorithms described in section 2 to support 16 bit floating point synaptic weights which should offer a significant performance improvement while not sacrificing the convenience of floating point programming.

In this paper we have only considered single-GPU simulations of circuit-scale SNN models. However, using supercomputer systems, models with up to a billion neurons can now be simulated (Jordan et al., 2018) and computational neuroscientists are beginning to use this capability to investigate the interactions between multiple circuit-scale models. For example, Schmidt et al. (2015) developed a model of the Macaque visual cortex consisting of 32 cortical areas, each modeled as a customized version of the model described in section 2.3. Even if such a model were implemented using half-precision floating point weights, a single GPU would not have enough memory to simulate it. However, systems such as the NVIDIA DGX-2 (NVIDIA Corporation, 2018c) are now available which contain several Tesla V100 GPUs, connected through a crossbar with a 900 GB s-1 bisection bandwidth. While GeNN does not currently target such multi-GPU systems, because all of their GPUs are connected to a single host system and are all mapped into its memory space, they maintain many of the advantages of the single-GPU simulations discussed in this paper. Beyond this scale, further parallelism could be achieved by using MPI to distribute GPU-accelerated simulations across multiple HPC nodes. While using MPI would lead to simulation becoming communication bound, as is currently the case with CPU simulations, fewer more powerful GPU-equipped nodes should reduce this problem as well as reducing power usage. Additionally, communication overheads could be reduced by using NVIDIA's GPUDirect (NVIDIA Corporation, 2018b) technology, allowing data to be transferred directly between remote GPUs via compatible network cards.

### 4.2. Comparison to Neuromorphic Systems

In section 3.3, we showed that our GPU simulations of the microcircuit model required less energy than those run on SpiNNaker. As van Albada et al. (2018) discuss, this poor energy efficiency comes from slowing SpiNNaker simulations down by a factor of 20 and only simulating 80 neurons on each core. However, because SpiNNaker is a software-programmable system, these limitations are not set in stone and Knight and Furber (2016) present some potential solutions to the underlying problems. Knight and Furber (2016) showed how models can be distributed more efficiently across a SpiNNaker machine and how Poisson background input could be directly injected into neurons to reduce the cost of incoming spike processing. Additionally, other software techniques we present in the context of GeNN such as the bitmask connectivity format and the parallel connectivity initialization would be potentially applicable to software-programmable neuromorphic systems such as SpiNNaker. Therefore, it seems possible that, purely through software improvements, SpiNNaker **could** simulate the microcircuit model with a much lower energy cost – perhaps closer to the 0.11 μJ per synaptic event measured by Sharp et al. (2012). Furthermore, by using more advanced power management techniques as well as reducing the manufacturing process size from 130 to 22 nm, the next generation SpiNNaker system aims to improve energy efficiency by a factor of 10 (Hoppner et al., 2017). Neuromorphic systems based on custom circuits rather than programmable CPUs still require much less energy. Digital system such as Intel's Loihi (Davies et al., 2018) or IBM's TrueNorth (Merolla et al., 2014) only require around 20 pJ per-synaptic event and analog systems such as Dynapse (Qiao et al., 2015) only require around 100 fJ per synaptic event. However, beside from SpiNNaker, only the Intel Loihi supports the degree of connectivity required to implement the microcircuit model.

Simulating the balanced random network model described in section 2.4 would be an even greater challenge for a neuromorphic system as, again, only SpiNNaker and Loihi would be able to support its high degree of connectivity. Additionally, while Loihi has a relatively flexible microcode-based “learning engine,” it does not directly support the operations required to calculate the *wij*^μ^ term in Equation (7). While several relatively complex synaptic plasticity rules have previously been implemented on SpiNNaker (Knight et al., 2016; Mikaitis et al., 2018b), these only required exponential decays and logarithms which could be implement using lookup tables, whereas, evaluating *wij*^μ^ would be likely to require a series expansion. Moise (2012) implemented several transcendental functions on SpiNNaker using series expansions and showed that they typically required in the order of 100 CPU cycles. Knight and Furber (2016) analyzed the performance of STDP processing on SpiNNaker and found that performing an additive weight update in response to a postsynaptic spike took around 31 CPU cycles. Therefore, adding the 100 extra CPU cycles required to evaluate *wij*^μ^ to this update, would be likely to severely reduce the STDP processing performance of SpiNNaker to the point that it would be unable to efficiently simulate this model. However, the next generation SpiNNaker system is likely to include bespoke accelerators to provide acceleration for *exp*(*x*) and *ln*(*x*) (Partzsch et al., 2017; Mikaitis et al., 2018a) which could be used to implement *wij*^μ^ as exp(μ·log(*wij*)).

### 4.3. Neurorobotics

Neurorobotics involves the development of robots with controllers inspired by the brain, allowing neural function to be studied in an embodied context. Neuro robots have been developed with controllers inspired by the mammalian Hippocampus (Krichmar et al., 2005) as well as the honey bee (Cope et al., 2016) and other insects (Blanchard et al., 2000). However, computational constraints have meant that these systems had to be operated either in simulation or their brain-inspired controllers had to be simulated externally to the robot. While using an external controller removes any constraints on the power and weight of the controller, it also introduces latency, meaning robots must operate slower. Additionally, communicating with an external controller typically means that a robot has to be “tethered” to a WiFi base station, restricting where it can operate.

The low power requirements and real-time performance of neuromorphic systems make them obvious candidates for building on-board neurorobotics controllers. However, in order to interface with the robots' hardware and convert sensor data into spike trains, these systems typically need to be accompanied by a standard CPU. For example, Kreiser et al. (2018) developed a path integration model on the Dynapse (Qiao et al., 2015) neuromorphic system which used a Parallela (Olofsson et al., 2015) board to interface with the robot and Hwu et al. (2017) developed a self-driving robot using a spiking convolutional neural network running on a TrueNorth NS1e development board which includes a Zynq SoC (Xilinx Inc, 2018). While both the Dynapse and TrueNorth systems have a negligible power consumption, the NS1e development board draws between 2 W to 3 W (Sawada et al., 2016) and the Parallela 5 W, somewhat out-weighing their theoretical advantages over embedded GPUs such as the Jetson TX2 which draws a maximum of 15 W (although Figure 12C suggests that, when simulating spiking neuron networks, the Jetson TX2 draws significantly less power).

Because SpiNNaker is built from programmable ARM cores, these can be repurposed for interfacing with robot hardware directly, for example using the interface board developed by Denk et al. (2013) which supports a variety of robots developed at the Technical University of Munich. However, the 48 chip SpiNNaker board used on the robot developed by Conradt et al. (2015) is around 10× larger than a Jetson TX2, restricting its use to large ground-based robots whereas the Jetson TX2 is small and light enough to be used on both ground and aerial robots. GeNN allows the development of SNN-based controllers that run on embedded GPUs such as the Jetson TX2 allowing them to control mobile robots of comparably small form factors with simulated brain circuits. While the simulations presented in this paper are too complex and are simulated on too small a simulation timestep for this to be possible, GPUs can simulate suitable models fast enough that, on average, simulation timesteps will complete in real-time. Although this does not guarantee that *every* simulation timestep will complete on time, neurorobotic controllers typically perform some form of low-pass filtering to convert spike trains to motor commands, meaning that some variability in the time taken to simulate each timestep is often acceptable. For example, the neurorobotic controller used by Kreiser et al. (2018) calculates motor commands from the number of spikes emitted in a 50 ms window (Milde et al., 2017).

This offers a very competitive alternative approach for neurorobotics research. For example, the “Brains on Board” project (www.brainsonboard.co.uk) is using GeNN on Jetson TX1 and TX2 GPUs to develop autonomous flying robots with navigational and learning abilities inspired by honeybees.

### 4.4. Interactive Simulation

As discussed in the introduction, one of the major uses of SNN simulations in computational neuroscience is for characterizing and exploring the subset of models' parameter spaces left under-constrained by experimental data.

In common with many other application areas, computational neuroscience simulations are typically performed in a non-interactive “batch” mode in which a simulation is started (either on a remote HPC system or locally) and some time later results are returned. The results of such simulations are then analyzed offline to determine whether a particular combination of parameters has resulted in a successful emulation of a brain circuit. However, it is difficult to determine what data will be required for this analysis ahead of time. Recording too much data requires large amounts of disk space and potentially slows down both, simulation and analysis. *Computational steering* (Parker et al., 1997) could be one solution to this problem—a technology that allows researchers to change the parameters of a running simulation as well as which state variables are being visualized.

With the development of large-scale models such as those discussed in the previous section, the need for approaches such as computational steering in computational neuroscience is becoming apparant. Nowke et al. (2018) developed a computational steering system for visualizing and steering NEST simulations. However, when running this system across a CPU-based HPC system, Nowke et al. found that its scalability was dictated by the amount of data that had to be transferred across the network at each simulation timestep. The next generation of supercomputer systems are being designed specifically to address these issues (Lippert and Orth, 2014). However, as discussed in section 4.3, GPUs are a more natural fit for this type of tight interaction between visualization and simulation as they exist within the host system's memory space, allowing data to be exchanged at the speed of the PCI express bus, rather than of an external network. Additionally, because CUDA can interact directly with graphics APIs such as OpenGL, some simple visualizations could be rendered without any interaction with the host computer's CPU at all.

## Author Contributions

JK and TN wrote the paper. TN is the original developer of GeNN. JK is currently the primary GeNN developer and was responsible for extending the code generation approach to the parallel initialization of networks. JK performed the experiments and the analysis of the results that are presented in this work.

## Data Availability Statement

All models, data and analysis scripts used for this study can be found in https://github.com/BrainsOnBoard/frontiers_genn_paper.

### Conflict of Interest Statement

The authors declare that the research was conducted in the absence of any commercial or financial relationships that could be construed as a potential conflict of interest.

## References

[B1] Auerswald E. Fontana C. (2018). Seven Segment Optical Character Recognition. Available online at: https://www.unix-ag.uni-kl.de/~auerswal/ssocr/

[B2] Awan M. G. Saeed F. (2016). GPU-arraysort: a parallel, in-place algorithm for sorting large number of arrays. Proceedings of the International Conference on Parallel Processing Workshops (Philadelphia, PA), 78–87. 10.1109/ICPPW.2016.27

[B3] Bi G. Q. Poo M. M. (1998). Synaptic modifications in cultured hippocampal neurons: dependence on spike timing, synaptic strength, and postsynaptic cell type. J. Neurosci. 18, 10464–10472. 10.1523/JNEUROSCI.18-24-10464.1998 9852584 PMC6793365

[B4] Blanchard M. Rind F. C. Verschure P. F. M. J. (2000). Collision avoidance using a model of the locust LGMD neuron. Robot. Auton. Syst. 30, 17–38. 10.1016/S0921-8890(99)00063-9

[B5] Brader J. M. Senn W. Fusi S. (2007). Learning real-world stimuli in a neural network with spike-driven synaptic dynamics. Neural Comput. 19, 2881–2912. 10.1162/neco.2007.19.11.2881 17883345

[B6] Braitenberg V. Schüz A. (2013). Cortex: Statistics and Geometry of Neuronal Connectivity. Berlin: Springer Science & Business Media.

[B7] Brette R. Goodman D. F. (2012). Simulating spiking neural networks on GPU. Netw. Comput. Neural Syst. 23, 167–182. 10.3109/0954898X.2012.730170 23067314

[B8] Brunel N. (2000). Dynamics of sparsely connected networks of excitatory and inhibitory spiking neurons. J. Comput. Neurosci. 8, 183–208. 10.1023/A:1008925309027 10809012

[B9] Brunel N. Hakim V. (1999). Fast global oscillations in networks of integrate-and-fire neurons with Low firing rates. Neural Comput. 11, 1621–1671. 10.1162/089976699300016179 10490941

[B10] Carnevale N. T. Hines M. L. (2006). The NEURON Book. Cambridge: Cambridge University Press 10.1017/CBO9780511541612

[B11] Chou T.-s. Kashyap H. J. Xing J. Listopad S. Rounds E. L. Beyeler M. (2018). CARLsim 4 : an open source library for large scale, biologically detailed spiking neural network simulation using heterogeneous clusters, in IEEE International Joint Conference on Neural Networks (IJCNN) (Rio de Janeiro), 1158–1165.

[B12] Clopath C. Büsing L. Vasilaki E. Gerstner W. (2010). Connectivity reflects coding: a model of voltage-based STDP with homeostasis. Nat. Neurosci. 13, 344–352. 10.1038/nn.2479 20098420

[B13] Conradt J. Galluppi F. Stewart T. C. (2015). Trainable sensorimotor mapping in a neuromorphic robot. Robot. Auton. Syst. 71, 60–68. 10.1016/j.robot.2014.11.004

[B14] Cope A. J. Richmond P. James S. S. Gurney K. Allerton D. J. (2017). SpineCreator: a graphical user interface for the creation of layered neural models. Neuroinformatics 15, 25–40. 10.1007/s12021-016-9311-z 27628934 PMC5306153

[B15] Cope A. J. Sabo C. Gurney K. Vasilaki E. Marshall J. A. R. (2016). A model for an angular velocity-tuned motion detector accounting for deviations in the corridor-centering response of the bee. PLoS Comput. Biol. 12:e1004887. 10.1371/journal.pcbi.1004887 27148968 PMC4858260

[B16] Davies M. Srinivasa N. Lin T.-h. Chinya G. Cao Y. Choday S. H. (2018). Loihi : a neuromorphic manycore processor with on-chip learning. IEEE Micro 30, 82–99. 10.1109/MM.2018.112130359

[B17] Davison A. P. Brüderle D. Eppler J. Kremkow J. Muller E. Pecevski D.. (2008). PyNN: a common interface for neuronal network simulators. Front. Neuroinform. 2:11. 10.3389/neuro.11.011.2008 19194529 PMC2634533

[B18] Denk C. Llobet-Blandino F. Galluppi F. Plana L. A. Furber S. Conradt J. (2013). Real-time interface board for closed-loop robotic tasks on the SpiNNaker neural computing system, in Artificial Neural Networks and Machine Learning–ICANN 2013. ICANN 2013. Lecture Notes in Computer Science, eds Mladenov V. Koprinkova-Hristova P. Palm G. Villa A. E. P. Appollini B. Kasabov N. (Berlin; Heidelberg: Springer), 467–474.

[B19] Devroye L. (2013). Non-uniform Random Variate Generation. New York, NY: Springer-Verlag.

[B20] Diamond A. Nowotny T. Schmuker M. (2016). Comparing neuromorphic solutions in action: implementing a bio-inspired solution to a benchmark classification task on three parallel-computing platforms. Front. Neurosci. 9:491. 10.3389/fnins.2015.00491 26778950 PMC4705229

[B21] Fidjeland A. K. Roesch E. B. Shanahan M. P. Luk W. (2009). NeMo: a platform for neural modelling of spiking neurons using GPUs, Proceedings of the International Conference on Application-Specific Systems, Architectures and Processors (Boston, MA), 137–144. 10.1109/ASAP.2009.24

[B22] Freedman D. Diaconis P. (1981). On the histogram as a density estimator: L 2 theory. Zeitschrift für Wahrscheinlichkeitstheorie und verwandte Gebiete 57, 453–476. 10.1007/BF01025868

[B23] Frenkel C. Legat J.-d. Bol D. (2018). A 0.086-mm^2^ 12.7-pJ/SOP 64k-synapse 256-neuron online-learning digital spiking neuromorphic processor in 28nm CMOS. IEEE Trans. Biomed. Circuits Syst. 10.1109/TBCAS.2018.2880425. [Epub ahead of print]. 30418919

[B24] Furber S. B. Galluppi F. Temple S. Plana L. A. (2014). The SpiNNaker Project. Proc. IEEE 102, 652–665. 10.1109/JPROC.2014.2304638

[B25] Galluppi F. Lagorce X. Stromatias E. Pfeiffer M. Plana L. A. Furber S. B.. (2014). A framework for plasticity implementation on the SpiNNaker neural architecture. Front. Neurosci. 8:429. 10.3389/fnins.2014.00429 25653580 PMC4299433

[B26] Garrido J. A. Carrillo R. R. Luque N. R. Ros E. (2011). Event and time driven hybrid simulation of spiking neural networks, in Advances in Computational Intelligence. IWANN 2011. Lecture Notes in Computer Science, eds Cabestany J. Rojas I. Joya G. (Berlin; Heidelberg: Springer), 554–561.

[B27] Gewaltig M.-O. Diesmann M. (2007). NEST (NEural Simulation Tool). Scholarpedia 2:1430 10.4249/scholarpedia.1430

[B28] Hanuschkin A. Kunkel S. Helias M. Morrison A. Diesmann M. (2010). A general and efficient method for incorporating precise spike times in globally time-driven simulations. Front. Neuroinform. 4:113. 10.3389/fninf.2010.00113 21031031 PMC2965048

[B29] Hoang R. V. Tanna D. Jayet Bray L. C. Dascalu S. M. Harris F. C. (2013). A novel CPU/GPU simulation environment for large-scale biologically realistic neural modeling. Front. Neuroinform. 7:19. 10.3389/fninf.2013.00019 24106475 PMC3788332

[B30] Hoppner S. Yan Y. Vogginger B. Dixius A. Partzsch J. Neumarker F. (2017). Dynamic voltage and frequency scaling for neuromorphic many-core systems, in 2017 IEEE International Symposium on Circuits and Systems (ISCAS) (Baltimore, MD: IEEE), 1–4. 10.1109/ISCAS.2017.8050656

[B31] Hwu T. Isbell J. Oros N. Krichmar J. (2017). A self-driving robot using deep convolutional neural networks on neuromorphic hardware. 2017 International Joint Conference on Neural Networks (IJCNN) (Anchorage, AK), 635–641. 10.1109/IJCNN.2017.7965912

[B32] Izhikevich E. M. (2007). Solving the distal reward problem through linkage of STDP and dopamine signaling. Cereb. Cortex 17, 2443–2452. 10.1093/cercor/bhl152 17220510

[B33] Jordan J. Ippen T. Helias M. Kitayama I. Sato M. Igarashi J. (2018). Extremely scalable spiking neuronal network simulation code: from laptops to exascale computers. Front. Neuroinform. 12:2 10.3389/fninf.2018.00002 29503613 PMC5820465

[B34] Knight J. C. Furber S. B. (2016). Synapse-centric mapping of cortical models to the SpiNNaker neuromorphic architecture. Front. Neurosci. 10:420. 10.3389/fnins.2016.00420 27683540 PMC5022244

[B35] Knight J. C. Tully P. J. Kaplan B. A. Lansner A. Furber S. B. (2016). Large-scale simulations of plastic neural networks on neuromorphic hardware. Front. Neuroanat. 10:37. 10.3389/fnana.2016.00037 27092061 PMC4823276

[B36] Kreiser R. Cartiglia M. Martel J. N. Conradt J. Sandamirskaya Y. (2018). A neuromorphic approach to path integration: a head-direction spiking neural network with vision-driven reset. In 2018 IEEE International Symposium on Circuits and Systems (ISCAS) (IEEE), 1–5.

[B37] Krichmar J. L. Seth A. K. Nitz D. A. Fleischer J. G. Edelman G. M. (2005). Spatial navigation and causal analysis in a brain-based device modeling cortical-hippocampal interactions. Neuroinformatics 3, 197–222. 10.1385/NI:3:3:197 16077159

[B38] Lippert T. Orth B. (2014). Supercomputing infrastructure for simulations of the human brain, in IET Computers & Digital Techniques (Cham), 198–212.

[B39] Markram H. (1997). Regulation of synaptic efficacy by coincidence of postsynaptic APs and EPSPs. Science 275, 213–215. 10.1126/science.275.5297.213 8985014

[B40] Merolla P. A. Arthur J. V. Alvarez-Icaza R. Cassidy A. S. Sawada J. Akopyan F.. (2014). A million spiking-neuron integrated circuit with a scalable communication network and interface. Science 345, 668–673. 10.1126/science.1254642 25104385

[B41] Micikevicius P. Narang S. Alben J. Diamos G. Elsen E. Garcia D. (2018). Mixed precision training, in Proceedings of the 6th International Conference on Learning Representations (Vancouver, BC).

[B42] Mikaitis M. Lester D. R. Shang D. Furber S. Liu G. Garside J. (2018a). Approximate fixed-point elementary function accelerator for the SpiNNaker-2 Neuromorphic Chip. in 2018 IEEE 25th Symposium on Computer Arithmetic (ARITH) (Amherst, MA: IEEE), 37–44.

[B43] Mikaitis M. Pineda García G. Knight J. C. Furber S. B. (2018b). Neuromodulated synaptic plasticity on the SpiNNaker neuromorphic system. Front. Neurosci. 12:105. 10.3389/fnins.2018.00105 29535600 PMC5835099

[B44] Milde M. B. Blum H. Dietmüller A. Sumislawska D. Conradt J. Indiveri G.. (2017). Obstacle avoidance and target acquisition for robot navigation using a mixed signal analog/digital neuromorphic processing system. Front. Neurorobot. 11:28. 10.3389/fnbot.2017.00028 28747883 PMC5507184

[B45] Moise M. (2012). A Fixed Point Arithmetic Library for SpiNNaker. Masters, The University of Manchester.

[B46] Moore S. W. Fox P. J. Marsh S. J. Markettos a. T. Mujumdar A. (2012). Bluehive - a field-programable custom computing machine for extreme-scale real-time neural network simulation, in 2012 IEEE 20th International Symposium on Field-Programmable Custom Computing Machines (Toronto, ON), 133–140.

[B47] Morrison A. Aertsen A. Diesmann M. (2007). Spike-timing-dependent plasticity in balanced random networks. Neural Comput. 19, 1437–1467. 10.1162/neco.2007.19.6.1437 17444756

[B48] Morrison A. Diesmann M. Gerstner W. (2008). Phenomenological models of synaptic plasticity based on spike timing. Biol. Cybernet. 98, 459–478. 10.1007/s00422-008-0233-1 18491160 PMC2799003

[B49] Nabavi S. Fox R. Proulx C. D. Lin J. Y. Tsien R. Y. Malinow R. (2014). Engineering a memory with LTD and LTP. Nature. 511, 348–352. 10.1038/nature13294 24896183 PMC4210354

[B50] Naylor M. Fox P. J. Markettos A. T. Moore S. W. (2013). Managing the FPGA memory wall: Custom computing or vector processing? in 2013 23rd International Conference on Field Programmable Logic and Applications, FPL 2013 - Proceedings (Porto).

[B51] Nowke C. Diaz-Pier S. Weyers B. Hentschel B. Morrison A. Kuhlen T. W.. (2018). Toward rigorous parameterization of underconstrained neural network models through interactive visualization and steering of connectivitygeneration. Front. Neuroinform. 12:32. 10.3389/fninf.2018.00032 29937723 PMC5992991

[B52] NVIDIA Corporation (2017). NVIDIA Tesla V100 GPU Architecture. White Paper.

[B53] NVIDIA Corporation (2018a). CUDA C Programming Guide.

[B54] NVIDIA Corporation (2018b). Developing a Linux Kernel Module Using RDMA for GPUDirect.

[B55] NVIDIA Corporation (2018c). DGX-2.

[B56] Olofsson A. Nordström T. Ul-Abdin Z. (2015). Kickstarting high-performance energy-efficient manycore architectures with Epiphany, Conference Record - Asilomar Conference on Signals, Systems and Computers (Pacific Grove, CA), 1719–1726.

[B57] Parker S. G. Johnson C. R. Beazley D. (1997). Computational steering software systems and strategies. IEEE Comput. Sci. Eng. 4, 50–59. 10.1109/99.641609

[B58] Partzsch J. Hoppner S. Eberlein M. Schuffny R. Mayr C. Lester D. R. Furber S. (2017). A fixed point exponential function accelerator for a neuromorphic many-core system, in Proceedings-IEEE International Symposium on Circuits and Systems (Baltimore, MD).

[B59] Pauli R. Weidel P. Kunkel S. Morrison A. (2018). Reproducing polychronization: a guide to maximizing the reproducibility of spiking network models. Front. Neuroinform. 12:46. 10.3389/fninf.2018.00046 30123121 PMC6085985

[B60] Potjans T. C. Diesmann M. (2014). The cell-type specific cortical microcircuit: relating structure and activity in a full-scale spiking network model. Cereb. Cortex 24, 785–806. 10.1093/cercor/bhs358 23203991 PMC3920768

[B61] Qiao N. Mostafa H. Corradi F. Osswald M. Stefanini F. Sumislawska D.. (2015). A reconfigurable on-line learning spiking neuromorphic processor comprising 256 neurons and 128K synapses. Front. Neurosci. 9:141. 10.3389/fnins.2015.00141 25972778 PMC4413675

[B62] Rall W. (1967). Distinguishing theoretical synaptic potentials computed for different soma-dendritic distributions of synaptic input. J. Neurophysiol. 30, 1138–1168. 10.1152/jn.1967.30.5.1138 6055351

[B63] Rittner P. Cleland T. A. (2016). Model definition and benchmarks for the Myriad parallel simulator, in Society for Neuroscience (Abstract) (San Diago, CA).

[B64] Rotter S. Diesmann M. (1999). Exact digital simulation of time-invariant linear systems with applications to neuronal modeling. Biol. Cybernet. 81, 381–402. 10.1007/s004220050570 10592015

[B65] Sawada J. Akopyan F. Cassidy A. S. Taba B. Debole M. V. Datta P. (2016). TrueNorth ecosystem for brain-inspired computing : scalable systems, software, and applications, in International Conference for High Performance Computing, Networking, Storage and Analysis, SC 16 (Salt Lake City, UT).

[B66] Schemmel J. Kriener L. Muller P. Meier K. (2017). An accelerated analog neuromorphic hardware system emulating NMDA- and calcium-based non-linear dendrites, Proceedings of the International Joint Conference on Neural Networks (Anchorage, AK), 2217–2226.

[B67] Schmidhuber J. (2015). Deep Learning in neural networks: an overview. Neural Netw. 61, 85–117. 10.1016/j.neunet.2014.09.003 25462637

[B68] Schmidt M. Bakker R. Shen K. Bezgin G. Hilgetag C.-C. Diesmann M. van Albada S. J. (2015). Full-density multi-scale account of structure and dynamics of macaque visual cortex. *arXiv:1511.09364*. Available online at: https://arxiv.org/abs/1511.09364

[B69] Seo J.-S. Brezzo B. Liu Y. Parker B. D. Esser S. K. Montoye R. K. (2011). A 45nm CMOS neuromorphic chip with a scalable architecture for learning in networks of spiking neurons, in 2011 IEEE Custom Integrated Circuits Conference (CICC) (San Jose, CA), 1–4.

[B70] Sharp T. Galluppi F. Rast A. Furber S. B. (2012). Power-efficient simulation of detailed cortical microcircuits on SpiNNaker. J. Neurosci. Methods 210, 110–118. 10.1016/j.jneumeth.2012.03.001 22465805

[B71] Sharp T. Petersen R. Furber S. B. (2014). Real-time million-synapse simulation of rat barrel cortex. Front. Neurosci. 8:131. 10.3389/fnins.2014.00131 24910593 PMC4038760

[B72] Song S. Miller K. D. Abbott L. F. (2000). Competitive Hebbian learning through spike-timing-dependent synaptic plasticity. Nat. Neurosci. 3, 919–926. 10.1038/78829 10966623

[B73] Stimberg M. Goodman D. F. M. Benichoux V. Brette R. (2014). Equation-oriented specification of neural models for simulations. Front. Neuroinform. 8:6. 10.3389/fninf.2014.00006 24550820 PMC3912318

[B74] Stimberg M. Goodman D. F. M. Nowotny T. (2018). Brian2genn: a system for accelerating a large variety of spiking neural networks with graphics hardware. bioRxiv. 10.1101/448050 PMC6962409 31941893

[B75] van Albada S. J. Helias M. Diesmann M. (2015). Scalability of asynchronous networks is limited by one-to-one mapping between effective connectivity and correlations. PLoS Comput. Biol. 11:e1004490. 10.1371/journal.pcbi.1004490 26325661 PMC4556689

[B76] van Albada S. J. Rowley A. G. Senk J. Hopkins M. Schmidt M. Stokes A. B.. (2018). Performance comparison of the digital neuromorphic hardware SpiNNaker and the neural network simulation software NEST for a full-scale cortical microcircuit Model. Front. Neurosci. 12:291. 10.3389/fnins.2018.00291 29875620 PMC5974216

[B77] Van Vreeswijk C. Abbott L. F. Bard Ermentrout G. (1994). When inhibition not excitation synchronizes neural firing. J. Comput. Neurosci. 1, 313–321. 10.1007/BF00961879 8792237

[B78] Villa O. Chavarria-Miranda D. Gurumoorthi V. Márquez A. Krishnamoorthy S. (2009). Effects of floating-point non-associativity on numerical computations on massively multithreaded systems, in Proceedings of Cray User Group Meeting (CUG) (Atlanta, GA).

[B79] Vitay J. Dinkelbach H. Ü. Hamker F. H. (2015). ANNarchy: a code generation approach to neural simulations on parallel hardware. Front. Neuroinform. 9:19. 10.3389/fninf.2015.00019 26283957 PMC4521356

[B80] Wang R. van Schaik A. (2018). Breaking Liebig ' s law : an advanced multipurpose neuromorphic engine. Front. Neurosci. 12:593 10.3389/fnins.2018.00593 30210278 PMC6123369

[B81] Xilinx Inc (2018). Zynq-7000 SoC.

[B82] Yavuz E. Turner J. Nowotny T. (2016). GeNN: a code generation framework for accelerated brain simulations. Sci. Rep. 6:18854. 10.1038/srep18854 26740369 PMC4703976

[B83] Yegenoglu A. Davison A. Holstein D. Muller E. Torre E. Hagen E. (2018). Elephant. Available online at: https://github.com/NeuralEnsemble/elephant/releases/tag/v0.5.0

